# Stochasticity Prevails but Differs: Tissue‐Specific Assembly of Gut Microbiomes Across Seasons in an Amphibian Model

**DOI:** 10.1002/ece3.73041

**Published:** 2026-02-03

**Authors:** Xiaowei Song, Yuanyuan Zhai, Mengyang Zhang, Jingyuan Guo, Benjun Guo, Chaolong Zhang, Jin Jin, Weiye Wang, Yuanping Xu, Bicheng Zhu, Xiangzhen Li

**Affiliations:** ^1^ Sichuan Provincial Engineering Research Center for Intelligent Tolerance Design and Measurement, College of Software Engineering Chengdu University of Information Technology Chengdu China; ^2^ Chengdu Institute of Biology Chinese Academy of Sciences Chengdu China; ^3^ College of Life Sciences Xinyang Normal University Xinyang China; ^4^ Engineering Research Center of Soil Remediation of Fujian Province University, College of Resources and Environment Fujian Agriculture and Forestry University Fuzhou China

**Keywords:** amphibian, assembly, ecological process, gut microbiota, hibernation, metamorphosis

## Abstract

Gut microbiota generally undergoes dynamic remodeling in concert with multifaceted self‐regulation of amphibian hosts during key life stages, such as metamorphosis and hibernation. However, the spatiotemporal dynamics of amphibian gut microbiomes across the lifecycle remain poorly understood. In this study, we applied 16S rRNA gene amplicon sequencing to characterize the gut microbiomes of cultivated Black‐spotted frog ( *Pelophylax nigromaculatus* ) across seasons. The gut microbiomes exhibited tissue‐specific succession, and structural discrepancies between gut regions fluctuated temporally. Both small‐ and large‐intestine microbiomes showed temporal decay patterns in abundance‐unweighted intercommunity indices, but not in abundance‐weighted indices. Compared with large‐intestine microbiomes, small‐intestine microbiomes were more randomized yet more centralized in terms of amplicon sequence variants, particularly within Proteobacteria (especially *Pseudomonas*). The alpha diversity of small‐intestine microbiomes was comparatively lower, and their taxonomic composition was more stable over time. We further elucidated the assembly mechanisms of gut microbiomes by systematically analyzing dominant driving factors, ecological processes, phylogenetic traits, source‐sink relationships, and co‐occurrence networks. Stochastic processes played a dominant role in gut microbiome assembly, while deterministic processes (e.g., habitat filtering and microbial interaction) contributed more strongly to large gut microbiomes than to small gut microbiomes. Overall, this study provides insights into the ecological dynamics and assembly mechanisms of amphibian gut microbiomes across the lifecycle and may inform targeted microbiome modification for amphibian breeding and conservation.

## Graphical abstract

Amphibian gut microbiomes exhibit a temporal‐decay pattern in abundance‐unweighted intercommunity indices rather than abundance‐weighted indices. Stochastic processes play a dominant role in the gut microbiome assembly. Deterministic processes contribute more to large‐intestine microbiomes than to small‐intestine microbiomes.

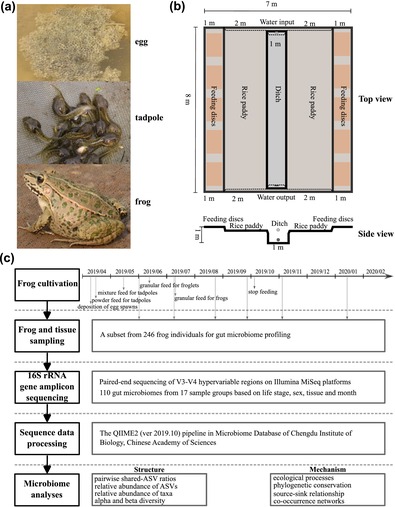

## 1 Introduction

Gut microbiota (i.e., microbial community) and amphibian hosts function as a holobiont through a stable yet dynamic symbiosis (Zilber‐Rosenberg and Rosenberg 2008). These microorganisms play essential roles in amphibian development, ecological adaptation, and health maintenance. For example, gut microbiota can promote metamorphosis and neurodevelopment by mediating the gut–liver and gut–brain axes (Emerson and Woodley 2024; Zhu et al. 2024). The maturation and homeostasis of the immune system are also likely supported by microbial metabolites of gut microbes, such as short‐chain fatty acids and secondary bile acids (Brown and Rudensky 2023; Tran et al. 2025). Conversely, dysbiosis of the gut microbiome (i.e., microorganisms and their theater of activity) may increase disease susceptibility and weaken resistance to parasites, particularly during early developmental stages (Berg et al. 2020; Knutie et al. 2017; Warne et al. 2019).

Amphibians undergo multifaceted self‐regulation across seasons, including changes in body morphology, dietary behavior, and physiological function during key life stages (Wang et al. 2025). For instance, larval organs such as intestines restructure extensively during metamorphosis to facilitate the transition from aquatic to territorial habitats. Adult amphibians enter hibernation to survive harsh environmental conditions, including prolonged food scarcity and cold exposure. Concurrently, gut microbiomes also remodel dramatically (Cao et al. 2023; Chai et al. 2018; Huang and Liao 2021; Park and Do 2024; Scalvenzi et al. 2021; Shi et al. 2023; Tong, Cui, et al. 2020; Tong et al. 2023; Wiebler et al. 2018; Zhang et al. 2019, 2020), driven by synergistic effects of environmental factors (e.g., habitat conditions) and host‐related factors (e.g., physiological status) (Levin et al. 2021; Martinez‐Guryn et al. 2019). However, gut microbiome succession across the amphibian lifecycle remains insufficiently studied (Scalvenzi et al. 2021; Shi et al. 2023; Tong, Cui, et al. 2020). In particular, temporal fluctuations of microbial communities in different intestinal regions have been largely overlooked (Scalvenzi et al. 2021; Zhang et al. 2019), limiting a comprehensive understanding of gut microbiomes across both temporal and spatial scales.

Beyond deterministic forces such as habitat filtering and microbial interactions, gut microbiome assembly can also be shaped by stochastic ecological processes, including dispersal and ecological drift (Kern et al. 2021; Zhou and Ning 2017). Our pilot studies have highlighted the importance of stochastic processes in shaping gut microbiomes of hibernating amphibians (Song et al. 2021, 2023). Nevertheless, most present studies of amphibian gut microbiomes have paid limited attention to stochasticity. This knowledge gap hinders a mechanistic understanding of gut microbiome assembly and its role in amphibian physiological adaptation.

**FIGURE 1:**
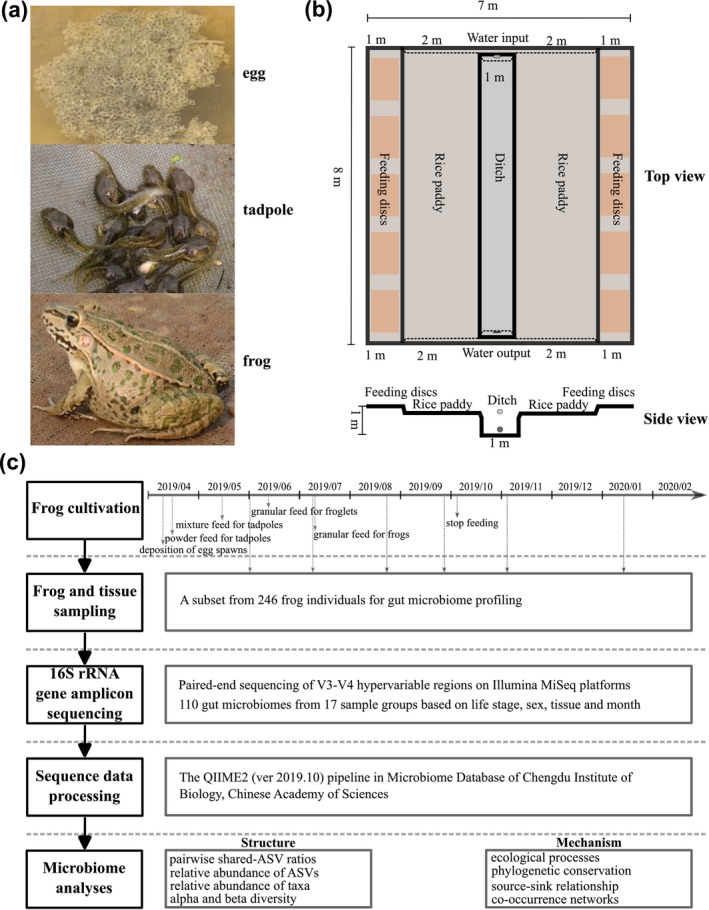
(a) Photographs of Black‐spotted frog ( *Pelophylax nigromaculatus* ) at three representative life stages. (b) Experimental field for rice‐frog co‐cultivation. (c) Research workflow in this study.

In this study, we aimed to characterize the spatiotemporal dynamics of amphibian gut microbiomes across seasons and to elucidate their underlying assembly mechanisms, with particular emphasis on stochastic processes. Cultivated Black‐spotted frog ( *Pelophylax nigromaculatus* ) was selected as the model organism because its living conditions and genetic backgrounds can be effectively controlled (Figure 1). Frog individuals were sampled successively over 6 months, and gut microbiomes were profiled using 16S rRNA gene amplicon sequencing. Microbial community structure was assessed using multiple diversity and compositional indices. Furthermore, we investigated gut microbiome assembly by systematically analyzing dominant driving factors, ecological processes, phylogenetic traits, source‐sink relationships, and co‐occurrence networks.

## 2 Materials and Methods

### 2.1 Animal Cultivation and Field Experiment

A rectangular field for rice‐frog (RF) co‐cultivation was established in January 2019 (Figure 1). The experimental field consisted of three main components: a ditch for water inflow and outflow, a rice paddy, and a feeding‐table area. The aquaculture water was pumped into the field from a nearby river. Cultivation procedures were manually adjusted to meet the requirements of frogs at different life stages, including hind‐limbed tadpoles (TH), fore‐limbed tadpoles (TF), froglets (FL), active frogs (FA), prehibernation frogs (PH), and mid‐hibernation frogs (MH). All animal cultivation followed local aquaculture regulations, and the experimental procedures were approved by the Animal Care and Use Committee in Xinyang Normal University (XFEC‐2018‐09).

### 2.2 Frog and Tissue Sampling

A total of 246 frog individuals were collected from the RF field over 6 months: June 1, 2019; July 7, 2019; August 24, 2019; September 27, 2019; November 4, 2019; and January 12, 2020. Randomly sampled frogs were euthanized by immersion in 95% ethanol (tadpoles and froglets) or by double pithing of the brain and spinal cord. Snout–vent length (SVL) and body mass were measured, and sex was determined by visual inspection of the gonads. The intestinal tissues were dissected under aseptic conditions and immediately stored at −20°C. The small gut (SG) and large gut (LG) were separately collected for each individual, except for tadpoles or froglets sampled in June, for which the whole gut (WG) was collected.

### 2.3 DNA Extraction and 16S rRNA Gene Amplicon Sequencing

To profile gut microbiomes, a subset of 75 frog individuals was selected for DNA extraction. Intestine tissues were rinsed with sterile water and opened using sterilized scissors under aseptic conditions. Total microbial genomic DNA was extracted using a modified phenol‐chloroform method (Song et al. 2018). After assessing DNA purity and concentration, 16S rRNA gene amplicon libraries targeting the V3–V4 hypervariable regions (primers 341F/805R) were constructed and sequenced using paired‐end sequencing on an Illumina MiSeq platform (Biobit Biotech Inc., China) (Song et al. 2021). In total, 110 gut microbiomes were obtained and categorized into 17 sample groups based on life stage, sex, tissue type, and sampling month (Tables 1 and S1). The sequencing data have been deposited in the National Microbiology Data Center under project number NMDC10020041.

**TABLE 1:** Sample sizes of 17 gut microbiome groups (*n* = 110), named using an integrated format of life stage, sex, tissue type, and sampling month.

Group	Sample size
TH‐U‐WG‐JUN	5
TF‐U‐WG‐JUN	6
FL‐U‐WG‐JUN	5
FA‐M‐SG‐JUL	9
FA‐M‐LG‐JUL	8
FA‐F‐SG‐JUL	5
FA‐F‐LG‐JUL	5
FA‐M‐SG‐AUG	8
FA‐M‐LG‐AUG	9
FA‐M‐SG‐SEP	10
FA‐M‐LG‐SEP	10
FA‐F‐SG‐SEP	5
FA‐F‐LG‐SEP	5
PH‐M‐SG‐NOV	6
PH‐M‐LG‐NOV	6
MH‐M‐SG‐JAN	4
MH‐M‐LG‐JAN	4

Abbreviations: AUG, August; F, female; FA, active frog; FL, froglet; JAN, January; JUL, July; JUN, June; LG, large gut; M, male; MH, mid‐hibernation; NOV, November; PH, pre‐hibernation; SEP, September; SG, small gut; TF, fore‐limbed tadpole; TH, hind‐limbed tadpole; U, unknown; WG, whole gut.

### 2.4 ASV Identification and Taxonomic Classification

Amplicon sequence variants (ASVs) were identified from the 110 gut microbiomes in this study, together with an additional 131 microbiomes that will be reported elsewhere. ASV inference was conducted using a QIIME2‐based pipeline (ver 2019.10) (Bolyen et al. 2019). Quality control included removal of sequences containing ambiguous bases, filtering by PHRED score (≥ 4), trimming to 200 bp, and denoising using the deblur algorithm (Amir et al. 2017). These ASVs were phylogenetically analyzed using MAFFT and FastTree (Katoh and Standley 2013; Price et al. 2010). Taxonomic annotation was performed using the classifier of the SILVA 16S rRNA database (ver 132) with a confidence threshold of 0.8 (Quast et al. 2012). The ASV table was filtered to retain ASVs with a total frequency ≥ 10, occurrence in at least two samples, exclusion of mitochondrial and chloroplast sequences, and inclusion of only bacterial and archaeal taxa. Two samples (IIIAS30 and IIIAS51) with insufficient sequencing depth were excluded from further analyses.

### 2.5 Structural Analyses of Gut Microbiomes

Structural analyses were performed in R (ver 4.4.0) using both unrarefied and rarefied datasets (R Core Team 2024). Major R packages included microeco, vegan, ape, magrittr, ggpubr, tidyr, and data.table (Bache and Wickham 2022; Barrett et al. 2024; Kassambara 2023; Liu et al. 2021; Oksanen et al. 2022; Paradis and Schliep 2019). For unrarefied data, ASV relative abundances and pairwise shared‐ASV ratios were calculated. Rarefaction was performed to a sequencing depth of 8868 (minimum depth among retained samples), after which alpha diversity (Observed ASVs, Shannon, Simpson, and Faith's phylogenetic diversity), beta diversity (Bray–Curtis, Jaccard, weighted and unweighted UniFrac), and taxonomic relative abundances (phylum, family, genus levels) were computed.

To examine temporal succession patterns, 14 group pairs were compared while controlling for tissue and sex effects. Tissue‐ and sex‐related differences were assessed using 11 pairs of contemporaneous samples. ASVs were classified into four abundance categories: None (0), Rare (0–0.001), Moderate (0.001–0.01), and Abundant (> 0.01). Temporal changes of ASVs were categorized as Decreased, Increased, or Unchanged based on Wilcoxon rank‐sum tests between temporally adjacent samples. Chi‐squared tests were used to evaluate differences in ASV categories and fluctuation types. The pairwise shared‐ASV ratios and alpha diversity were visualized using boxplots and tested with Wilcoxon rank‐sum tests. Beta diversity was visualized using boxplots and principal coordinates analysis (PCoA), and assessed using Wilcoxon tests and PERMANOVA. Taxonomic composition was visualized with alluvial plots, and differential taxa were identified using LEfSe and random forest analyses (Liu et al. 2021; Segata et al. 2011). The filtering thresholds of taxonomic relative abundance, logarithmic LDA score, and mean Gini decrease were set as 0.001, 4, and 0.5. Statistical significance was set at *p* < 0.05 unless otherwise stated, and *p* values were adjusted using the Benjamini–Hochberg FDR method.

Temporal decay was evaluated by calculating Spearman correlations between structural dissimilarities and temporal intervals (Shade et al. 2013). The analyses were conducted separately for SG and LG microbiomes, excluding female samples. Structural divergence between SG and LG microbiomes was also assessed across months using male samples only.

### 2.6 Assembly Mechanism Analyses of Gut Microbiomes

Variance partitioning analysis (VPA) was applied to evaluate the explanatory power of factors on structural variation of gut microbiomes (Legendre 2008; Oksanen et al. 2022). Separate VPAs were performed for three sets of factors, each including three dominant factors (tissue, month, life stage) and one subdominant factor (sex, SVL, or temperature). Factor significance was assessed using the “anova.cca” function of the vegan package.

Ecological processes governing gut microbiome assembly were inferred using phylogenetic bin‐based null model analysis methods, that is, “icamp.cm”, “icamp.bins” and “icamp.cate” functions implemented in the iCAMP package (ver 1.5.12) (Ning et al. 2020). ASVs were grouped into phylogenetic bins with “bin.size.limit = 48”. Ecological processes were classified as homogeneous selection, heterogeneous selection, homogenizing dispersal, dispersal limitation, and drift based on βMNTD_RC and RCbray values.

Phylogenetic structure of gut microbiomes was evaluated using confidence indices (cMPD and cMNTD) (Ning et al. 2020). Phylogenetic conservation in the niche adaptation of gut microbiomes was assessed by examining whether explanatory powers of factors had upward or stable trends across taxonomic levels (Lu et al. 2016). The structural variation data were allocated to three sets of factors in a similar manner as above VPAs.

Source–sink relationships were inferred using FEAST (version 0.1.0) to quantify contributions from temporal transmission, spatial dispersal, and unknown sources (Shenhav et al. 2019).

Co‐occurrence networks were constructed using the microeco package (ver 1.6.0) for eight gut microbiome groups (Liu et al. 2021). Spearman correlations were calculated among ASVs, and network modules were partitioned using the Louvain method. The network topology and robustness were compared using the meconetcomp package (ver 0.6.1) (Liu et al. 2023).

## 3 Results

### 3.1 The Dynamics of Gut Microbiomes Across Months or Life Stages

**FIGURE 2:**
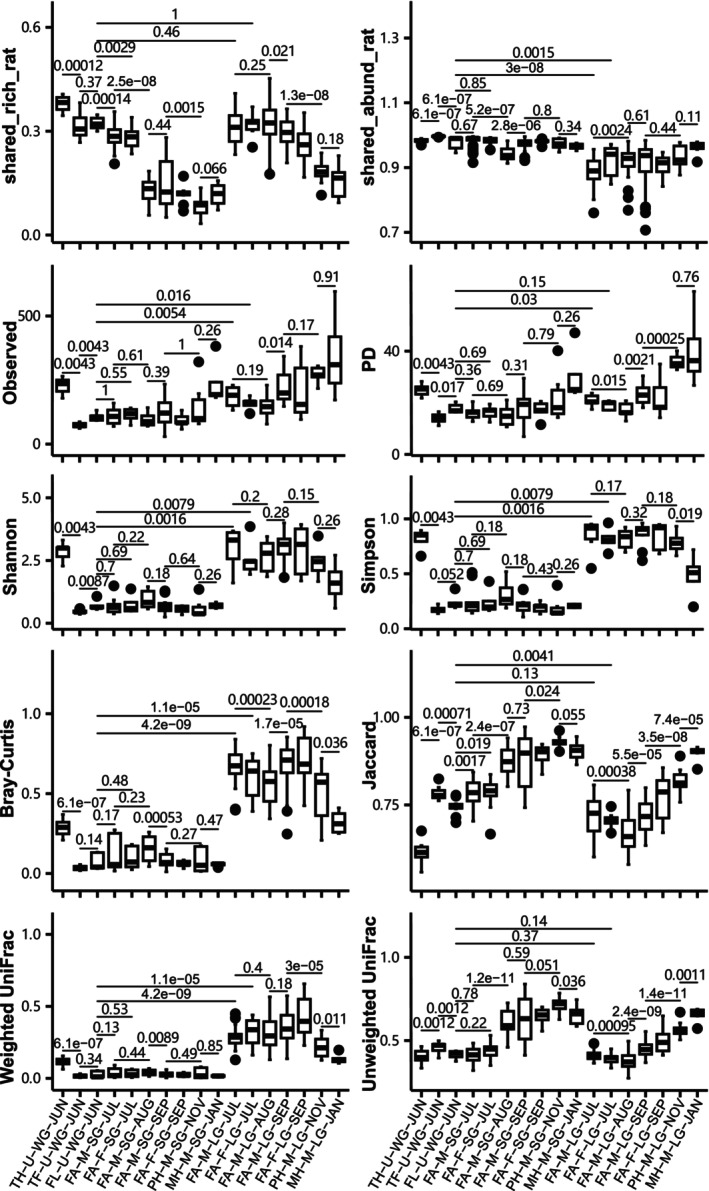
Structural comparisons across months or life stages using pairwise shared‐ASV ratios, alpha diversity, and beta diversity indices. The shared_abund_rat and shared_rich_rat represent abundance‐weighted and abundance‐unweighted pairwise shared‐ASV ratios, respectively. The significance level for Wilcoxon rank sum tests was set to 0.05.

**FIGURE 3:**
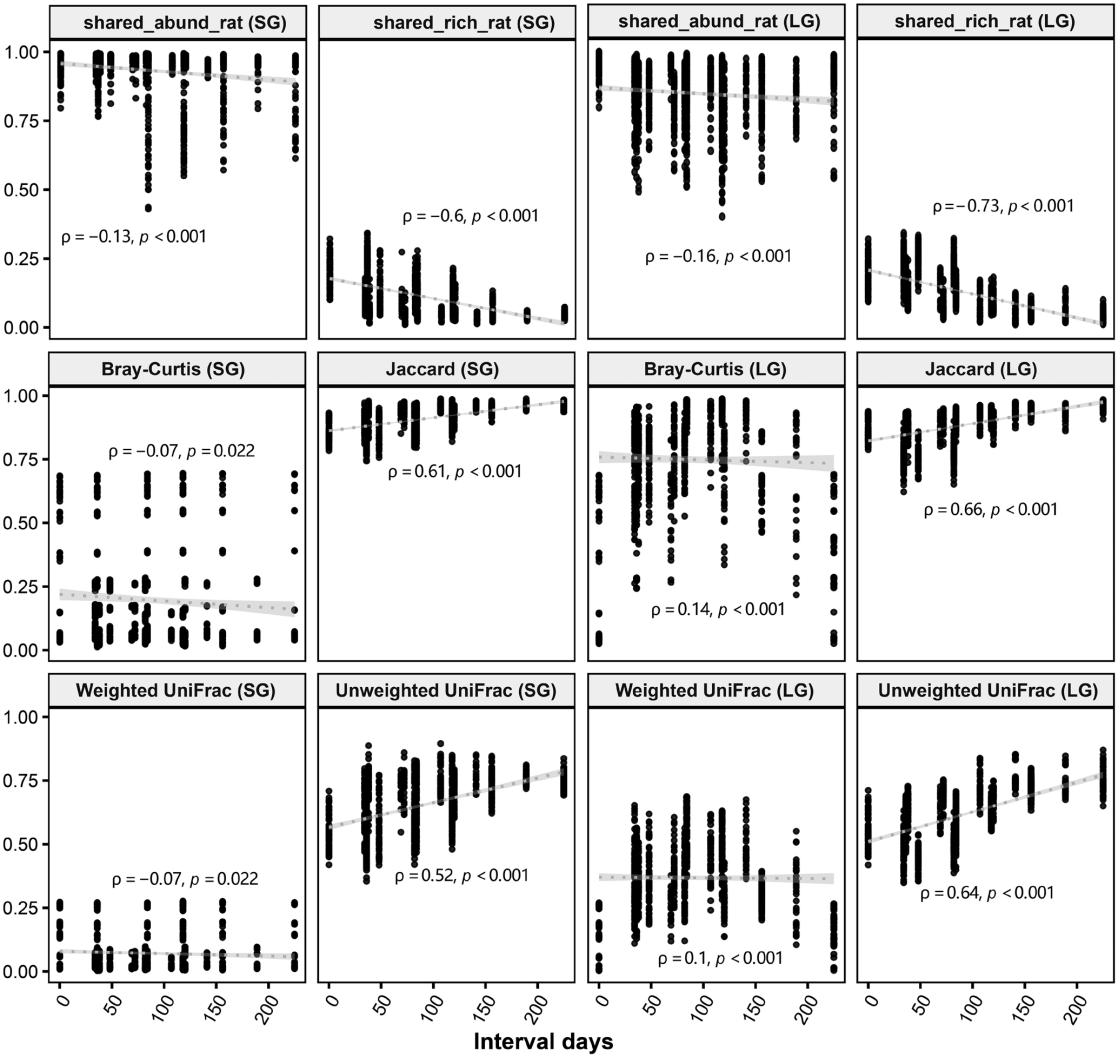
Spearman correlations between inter‐group indices and their interval days. SG and LG microbiomes were analyzed separately, excluding female samples. Linear regression lines with 95% confidence intervals are shown in gray.

Most ASVs were rare or absent in gut microbiome groups and remained stable in relative abundance during succession (Tables 2 and 3). However, almost all temporally adjacent groups showed different proportions of ASV categories and ASV fluctuation types (Tables S2 and S3). Gut microbiomes exhibited complex succession trends in terms of pairwise shared‐ASV ratios, alpha diversity, and beta diversity indices (Figure 2). The abundance‐unweighted pairwise shared‐ASV ratios and beta diversity indices gradually decreased and increased across months, respectively, whereas abundance‐weighted indices remained comparatively stable. Furthermore, the dynamics of gut microbiomes was characterized by significant tissue specificity (Figures 2 and S1, Table 4). For instance, the abundance‐weighted indices of LG microbiomes presented greater variation than those of SG microbiomes. Significant differences were detected in abundance‐weighted indices of temporally adjacent LG microbiomes, but not in SG microbiomes. LG microbiomes displayed greater temporal fluctuation of taxonomic composition than SG microbiomes (Figure S2). Proteobacteria and Firmicutes were the most dominant taxa in SG and LG microbiomes from July through November, respectively. Several genera markers were consistently identified by LEfSe and random forest methods across four pairs of temporally adjacent groups: (1) TH‐U‐WG‐JUN (*Mycobacterium*) and TF‐U‐WG‐JUN (*Pseudomonas*); (2) FA‐M‐LG‐JUL (*Pseudomonas*) and FA‐M‐LG‐AUG (*Cetobacterium*); (3) FA‐M‐LG‐AUG (*Cetobacterium*, *Escherichia−Shigella*) and FA‐M‐LG‐SEP (*Akkermansia*); (4) FA‐M‐LG‐SEP (*Bacteroides*, *Erysipelatoclostridium*) and PH‐M‐LG‐NOV (*Pseudomonas*). Temporal‐decay phenomena occurred in the abundance‐unweighted between‐group indices of SG and LG microbiomes (Figure 3), with significant negative Spearman correlations observed between unweighted indices and the interval days.

**TABLE 2:** Proportions of ASV categories in SG and LG microbiomes across months or life stages.

ASV number	Group	*N* (%)	*R* (%)	*M* (%)	*A* (%)	*p* (*Χ* ^2^)
5274 (SG microbiomes)	TH‐U‐WG‐JUN	83.52	15.28	0.93	0.27	< 0.001
	TF‐U‐WG‐JUN	89.29	10.60	0.06	0.06	
	FL‐U‐WG‐JUN	89.29	10.43	0.23	0.06	
	FA‐M‐SG‐JUL	85.93	13.84	0.17	0.06	
	FA‐M‐SG‐AUG	90.77	8.84	0.32	0.08	
	FA‐M‐SG‐SEP	80.32	19.45	0.19	0.04	
	PH‐M‐SG‐NOV	48.84	51.02	0.09	0.04	
	MH‐M‐SG‐JAN	60.50	39.40	0.06	0.04	
6294 (LG microbiomes)	TH‐U‐WG‐JUN	86.19	12.81	0.78	0.22	< 0.001
	TF‐U‐WG‐JUN	91.02	8.88	0.05	0.05	
	FL‐U‐WG‐JUN	91.02	8.74	0.19	0.05	
	FA‐M‐LG‐JUL	84.40	14.19	1.10	0.32	
	FA‐M‐LG‐AUG	87.75	11.17	0.84	0.24	
	FA‐M‐LG‐SEP	77.30	21.58	0.84	0.29	
	PH‐M‐LG‐NOV	58.61	40.48	0.75	0.16	
	MH‐M‐LG‐JAN	43.96	55.54	0.40	0.10	

*Note:* WG microbiomes were integrated in the analyses of SG and LG microbiomes, but female samples were excluded. The characters of *N*, *R*, *M* and *A* represent four ASV categories, i.e., None, Rare, Moderate and Abundant. Chi‐square (*Χ* ^2^) tests were applied to assess independence between temporal groups and ASV categories.

**TABLE 3:** Proportions of ASV fluctuation types in SG and LG microbiomes across months or life stages.

ASV number	Group	*D* (%)	*I* (%)	*U* (%)	*p* (*Χ* ^2^)
5274 (SG microbiomes)	TH‐U‐WG‐JUN|TF‐U‐WG‐JUN	4.04	0.66	95.30	< 0.001
	TF‐U‐WG‐JUN|FL‐U‐WG‐JUN	0.28	1.12	98.60	
	FL‐U‐WG‐JUN|FA‐M‐SG‐JUL	1.02	0.34	98.63	
	FA‐M‐SG‐JUL|FA‐M‐SG‐AUG	0.97	0.25	98.79	
	FA‐M‐SG‐AUG|FA‐M‐SG‐SEP	0.21	0.30	99.49	
	FA‐M‐SG‐SEP|PH‐M‐SG‐NOV	0.28	2.07	97.65	
	PH‐M‐SG‐NOV|MH‐M‐SG‐JAN	0.00	0.78	99.22	
6294 (LG microbiomes)	TH‐U‐WG‐JUN|TF‐U‐WG‐JUN	3.38	0.56	96.06	< 0.001
	TF‐U‐WG‐JUN|FL‐U‐WG‐JUN	0.24	0.94	98.82	
	FL‐U‐WG‐JUN|FA‐M‐LG‐JUL	1.46	1.67	96.87	
	FA‐M‐LG‐JUL|FA‐M‐LG‐AUG	1.26	0.76	97.98	
	FA‐M‐LG‐AUG|FA‐M‐LG‐SEP	0.76	2.57	96.66	
	FA‐M‐LG‐SEP|PH‐M‐LG‐NOV	1.59	4.53	93.88	
	PH‐M‐LG‐NOV|MH‐M‐LG‐JAN	0.29	1.13	98.59	

*Note:* WG microbiomes were integrated in the analyses of SG and LG microbiomes, but female samples were excluded. The characters of D, I, and U represent three ASV fluctuation types, that is, Decreased, Increased, and Unchanged. Chi‐square (*Χ* ^2^) tests were applied to assess independence between temporal groups and ASV fluctuation types.

### 3.2 The Structural Divergence Between Small and Large Gut Microbiomes

**FIGURE 4:**
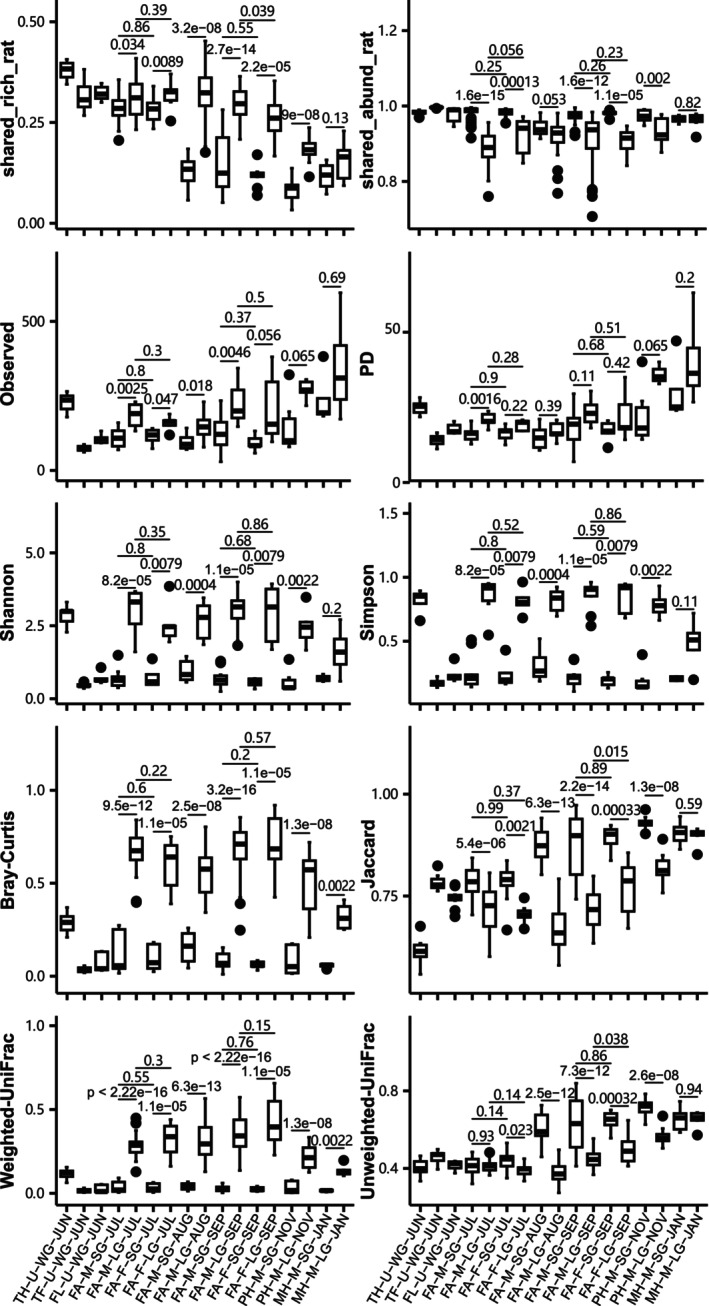
Structural comparisons between tissues and between sexes using pairwise shared‐ASV ratios, alpha diversity, and beta diversity indices. The significance level for Wilcoxon rank sum tests was set to 0.05.

Contemporary SG and LG microbiomes, except those in January, showed significant structural differences in ASV categories, ASV fluctuation types, pairwise shared‐ASV ratios, alpha diversity, and beta diversity indices (Figure 4, Tables S5 and S6). By contrast, differences between sexes were weak or absent. Specifically, alpha diversity of SG microbiomes was lower than that of LG microbiomes. The structural differences between tissues were opposite for the abundance‐unweighted and abundance‐weighted intercommunity indices. SG and LG microbiomes initially diverged and later converged during succession (Figures S1 and S3). Taxonomic composition differed significantly between tissues, but not between sexes. Tissue‐associated genera markers consistently identified by LEfSe and random forest analyses included: (1) FA‐F‐SG‐JUL (*Pseudomonas*) and FA‐F‐LG‐JUL (*endosymbiont ‘TC1’ of Trimyema compressum*); (2) FA‐M‐SG‐JUL (*Pseudomonas*) and FA‐M‐LG‐JUL (*endosymbiont ‘TC1’ of Trimyema compressum*, *Erysipelatoclostridium* and *Ruminococcaceae_UCG−014*); (3) FA‐M‐SG‐AUG (*Pseudomonas*) and FA‐M‐LG‐AUG (*Cetobacterium*); (4) FA‐M‐SG‐SEP (*Pseudomonas*) and FA‐M‐LG‐SEP (*Breznakia*, *Akkermansia* and *Erysipelatoclostridium*); (5) PH‐M‐SG‐NOV (*Pseudomonas*) and PH‐M‐LG‐NOV (*endosymbiont ‘TC1’ of Trimyema compressum*).

### 3.3 The Dominant Factors for the Structural Variation of Gut Microbiomes

**FIGURE 5:**
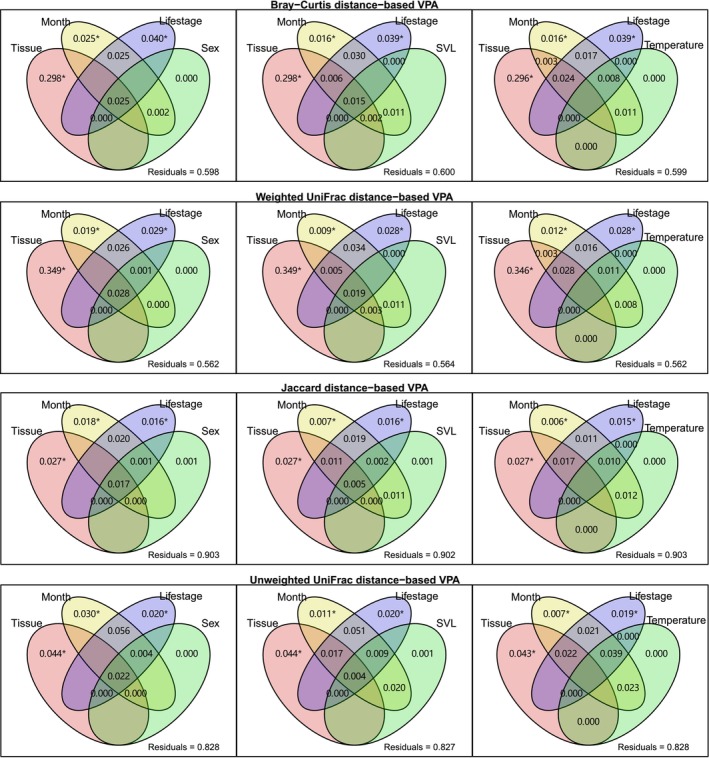
Variance partitioning analysis (VPA) on beta diversity indices of gut microbiomes for three groups of factors. Each group included three dominant factors (Tissue, Month, Life stage) and one subdominant factor (Sex, SVL, or Temperature). Negative values are not shown. *The conditional significance of the deterministic factor < 0.05.

The main factors explaining structural variation of gut microbiomes were tissue, month, and life stage, rather than sex, SVL, or temperature (Figure 5). The host and environmental factors had greater explanatory power for abundance‐weighted structural variation than for abundance‐unweighted variation. For example, tissue, month, life stage, and sex explained 40.2% of Bray‐Curtis and 43.8% of weighted UniFrac distances, compared to only 9.7% of Jaccard and 17.2% of unweighted UniFrac distances. Nevertheless, most structural variation remained unexplained by these factors.

### 3.4 The Ecological Processes to the Assembly of Gut Microbiomes

**FIGURE 6:**
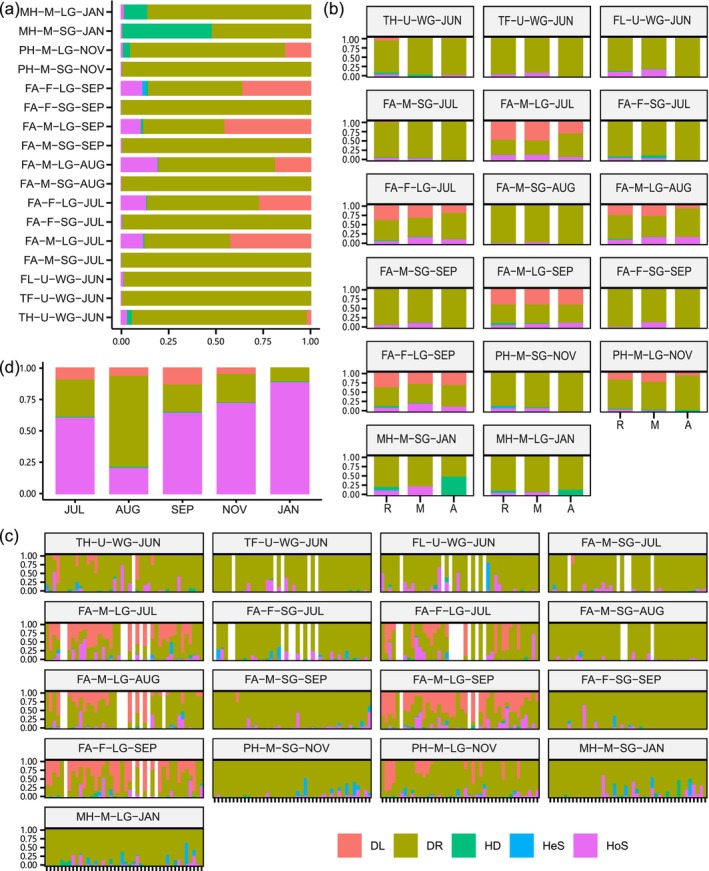
Relative importance of ecological processes deduced with bin‐based null model of R package iCAMP (ver 1.5.12) for: (a) gut microbiome groups; (b) ASV categories; (c) bins; (d) between male SG and LG microbiomes. A, abundant; DL, dispersal limitation; DR, drift; HD, homogenizing dispersal; HeS, heterogeneous selection; HoS, homogeneous selection; M, moderate; R, rare.

Gut microbiome assembly was dominated by stochastic ecological processes, with the relative importance ranging from 80.43% to 99.71% (Figure 6a). Ecological processes varied spatiotemporally and showed clear heterogeneity between ASV categories and bins (Figure 6b,c). For active frogs, drift accounted for almost all ecological processes in SG microbiomes, whereas dispersal limitation and homogeneous selection contributed substantially to LG microbiomes. During hibernation, dispersal limitation and homogeneous selection decreased dramatically in LG microbiomes, while homogenizing dispersal increased, especially in MH. Ecological processes between SG and LG microbiomes fluctuated significantly from July through January (Figure 6d). Homogeneous selection accounted for the smallest proportion in August (20.99%) and the largest in January (89.07%). Dispersal limitation was detected from July through November but was absent in January.

### 3.5 The Phylogenetic Traits of Gut Microbiomes

**FIGURE 7:**
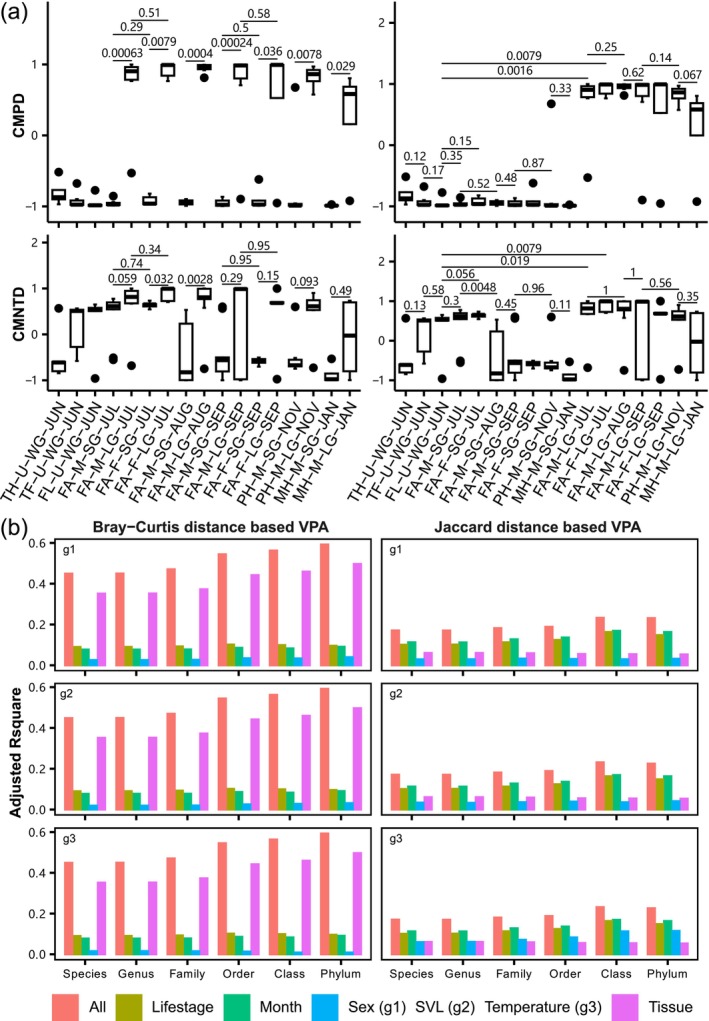
(a) Wilcoxon rank sum tests on the CMPD and CMNTD indices of contemporary and temporally adjacent gut microbiomes. The significance level was set to 0.05. (b) The adjusted *R*‐Squares of three groups of factors in variance partitioning analysis (VPA) across different taxonomic levels. g1, Tissue, Month, Life stage and Sex factors; g2, Tissue, Month, Life stage and SVL factors; g3, Tissue, Month, Life stage and Temperature factors.

Gut microbiomes exhibited variable phylogenetic convergence across space and time (Figure 7). All taxa showed high phylogenetic divergence during metamorphosis, whereas nearest taxa in TF and FL displayed greater convergence. SG and LG microbiomes generally exhibited divergent and convergent phylogenetic traits, respectively. Nearest taxa of SG microbiomes in July retained convergent traits of TF and FL, which differed from subsequent months. During hibernation, LG microbiomes gradually shifted from high convergence to near randomization. In addition, phylogenetic conservation signals were detected for gut microbiomes in the niche adaptation, particularly in tissue‐associated niches (Figure 7).

### 3.6 The Source‐Sink Relationships of Gut Microbiomes

Source‐sink relationships among gut microbiomes varied spatiotemporally (Table 4). During metamorphosis, TH sources contributed minimally (~1%) to TF sinks, whereas TF sources contributed the largest proportion (~89%) to FL sinks. Temporal transmission contributed far more than spatial dispersal to SG microbiomes. For instance, SG microbiomes in July mainly derived from TF (~61%) and FL (~29%), rather than contemporary LG microbiomes (~6%). In LG microbiomes, contributions from spatial dispersal and temporal transmission fluctuated across months. LG microbiomes in July were primarily contributed by contemporary SG (~49%) and unknown sources (~43%). LG microbiomes in August and September derived mainly from counterparts in the previous month (~55% and ~80%), with minimal spatial dispersal (< 1% and ~5%). During hibernation, the contribution of spatial dispersal to LG microbiomes increased (~26% in November and ~22% in January).

**TABLE 4:** Proportions of gut microbiomes contributed from three sources: previous month or life stage, contemporary different tissue, and unknown.

Sinks	Sources	Proportions (Mean ± SD)
TF‐U‐WG‐JUN	TH‐U‐WG‐JUN	0.010 ± 0.002
	Unknown	0.990 ± 0.002
FL‐U‐WG‐JUN	TH‐U‐WG‐JUN	0.032 ± 0.006
	TF‐U‐WG‐JUN	0.890 ± 0.058
	Unknown	0.078 ± 0.058
FA‐M‐LG‐JUL	TH‐U‐WG‐JUN	0.015 ± 0.024
	TF‐U‐WG‐JUN	0.021 ± 0.054
	FL‐U‐WG‐JUN	0.042 ± 0.047
	FA‐M‐SG‐JUL	0.491 ± 0.162
	Unknown	0.431 ± 0.179
FA‐M‐SG‐JUL	TH‐U‐WG‐JUN	0.002 ± 0.001
	TF‐U‐WG‐JUN	0.610 ± 0.120
	FL‐U‐WG‐JUN	0.290 ± 0.070
	FA‐M‐LG‐JUL	0.062 ± 0.129
	Unknown	0.036 ± 0.074
FA‐M‐LG‐AUG	FA‐M‐SG‐JUL	0.002 ± 0.002
	FA‐M‐LG‐JUL	0.554 ± 0.160
	FA‐M‐SG‐AUG	0.006 ± 0.003
	Unknown	0.438 ± 0.161
FA‐M‐SG‐AUG	FA‐M‐SG‐JUL	0.673 ± 0.323
	FA‐M‐LG‐JUL	0.001 ± 0.002
	FA‐M‐LG‐AUG	0.012 ± 0.009
	Unknown	0.314 ± 0.324
FA‐M‐LG‐SEP	FA‐M‐SG‐AUG	0.001 ± 0.001
	FA‐M‐LG‐AUG	0.801 ± 0.208
	FA‐M‐SG‐SEP	0.054 ± 0.087
	Unknown	0.144 ± 0.149
FA‐M‐SG‐SEP	FA‐M‐SG‐AUG	0.960 ± 0.029
	FA‐M‐LG‐AUG	0.002 ± 0.002
	FA‐M‐LG‐SEP	0.017 ± 0.015
	Unknown	0.022 ± 0.014
FA‐M‐LG‐NOV	FA‐M‐SG‐SEP	0.094 ± 0.055
	FA‐M‐LG‐SEP	0.430 ± 0.110
	FA‐M‐SG‐NOV	0.262 ± 0.126
	Unknown	0.214 ± 0.176
FA‐M‐SG‐NOV	FA‐M‐SG‐SEP	0.853 ± 0.173
	FA‐M‐LG‐SEP	0.020 ± 0.048
	FA‐M‐LG‐NOV	0.012 ± 0.008
	Unknown	0.115 ± 0.177
FA‐M‐LG‐JAN	FA‐M‐SG‐NOV	0.273 ± 0.074
	FA‐M‐LG‐NOV	0.284 ± 0.170
	FA‐M‐SG‐JAN	0.221 ± 0.116
	Unknown	0.221 ± 0.209
FA‐M‐SG‐JAN	FA‐M‐SG‐NOV	0.774 ± 0.143
	FA‐M‐LG‐NOV	0.003 ± 0.001
	FA‐M‐LG‐JAN	0.089 ± 0.016
	Unknown	0.134 ± 0.131

### 3.7 The Co‐Occurrence Networks of Gut Microbiomes

**FIGURE 8:**
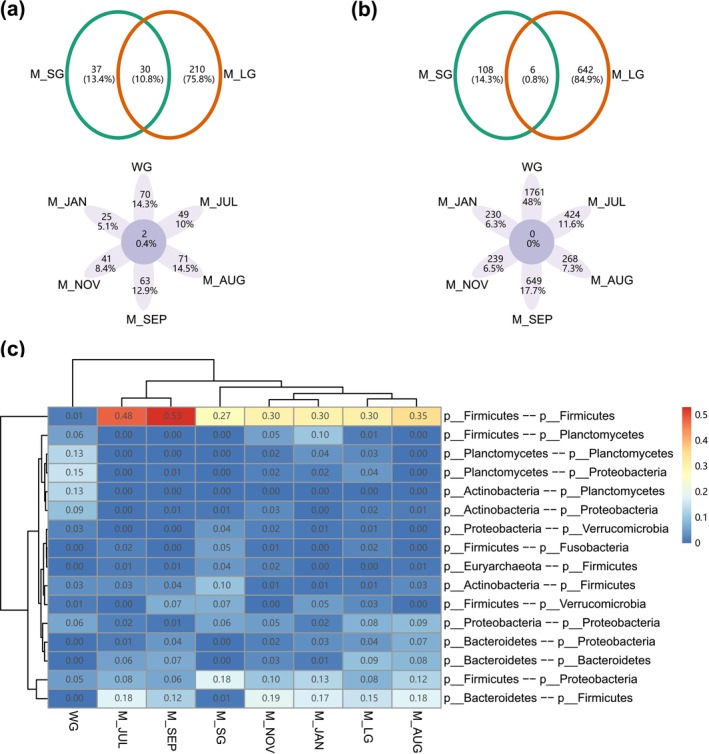
Comparisons of co‐occurrence networks across eight gut microbiome groups: (a) overlapping nodes; (b) overlapping edges; (c) taxonomic composition of positive edges. M_AUG, male gut microbiomes in August; M_JAN, male gut microbiomes in January; M_JUL, male gut microbiomes in July; M_LG, male large gut microbiomes; M_NOV, male gut microbiomes in November; M_SEP, male gut microbiomes in September; M_SG, male small gut microbiomes; WG, whole gut microbiomes in June.

Co‐occurrence networks exhibited significant temporal dynamics and tissue dependence (Figure 8 and Table S7). Overlapping nodes and edges between SG and LG networks were low (10.8% and 0.8%). Across 6 months, the networks shared 2 nodes (0.4%) and no edges. Many key nodes displayed temporal fluctuations or tissue‐specific differences (Table S8). For example, four key nodes in the WG network corresponded to genus markers of TH‐U‐WG‐JUN and TF‐U‐WG‐JUN, that is, *Mycobacterium* and *Pseudomonas*. The July network included six key nodes associated with genus markers of FA‐M‐SG‐JUL and FA‐M‐LG‐JUL, that is, *Pseudomonas*, *endosymbiont ‘TC1’ of Trimyema compressum*, *Erysipelatoclostridium*, and *Ruminococcaceae_UCG−014*. Positive network edges showed divergent taxonomic composition (Figure 8). For example, the June network had more edges associated with Actinobacteria (~25%) and Planctomycetes (~45%) than Firmicutes (~15%), whereas subsequent months had more edges associated with Firmicutes. LG network possessed more positive edges associated with Bacteroidetes than SG network. The network during metamorphosis showed greater robustness than those in later months (Figure S4), and the LG network was more robust than the SG network.

## 4 Discussion

Amphibian gut microbiomes undergo adaptive restructuring in response to dramatic changes of environmental and host factors across the lifecycle (Cao et al. 2023; Chai et al. 2018; Huang and Liao 2021; Park and Do 2024; Scalvenzi et al. 2021; Shi et al. 2023; Tong, Cui, et al. 2020; Tong et al. 2023; Wang et al. 2025; Wiebler et al. 2018; Zhang et al. 2019, 2020). Seasonal dynamics of gut microbiomes represent a widespread ecological strategy among animals, including insects (Fluch et al. 2024; Hendrycks et al. 2025), fish (Xu et al. 2024), and mammals (Fan et al. 2022). Here we also depict the complex gut microbiome succession of cultivated Black‐spotted frog across seasons. However, the underlying assembly mechanisms vary depending on host phylogeny, physiological traits, and ecological niche. For example, deterministic and stochastic processes play a considerable role in shaping the gut microbiomes of cucurbit‐feeding fruit flies (Hendrycks et al. 2025), while deterministic processes dominate in gut microbiota assembly of cold‐water fish (Xu et al. 2024). By contrast, stochastic processes play dominant roles in shaping frog gut microbiomes, although deterministic processes, particularly for LG microbiomes, remain significant. Structural divergence between SG and LG microbiomes appears to be driven largely by fluctuations of homogeneous selection and dispersal limitation.

During metamorphosis, amphibian larva experience body reconstruction for the adaptation from aquatic to territorial habitats (Hourdry et al. 1996). Once tadpoles enter the forelimb phase, they cease feeding, and their tails are absorbed. The intestines transform from a fish‐like form (long and undivided) into terrestrial form (shortened with enhanced differentiation) (Ishizuya‐Oka 2007). Concurrently, gut microbiomes shift from fish‐like to amniote‐like composition (Kohl et al. 2013). In line with previous studies (Chai et al. 2018; Shi et al. 2023; Tong, Cui, et al. 2020), species richness, evenness, and phylogenetic diversity decrease from TH to TF, and stabilize in FL. Compared to TH microbiomes, TF and FL microbiomes are more randomized, with core ASVs more centralized. Proteobacteria (especially *Pseudomonas*) accounts for a greater proportion in TF and FL, while other phyla such as Actinobacteria and Planctomycetes decrease. Similarly, Actinobacteria is enriched in the gut microbiomes of other anurans during early metamorphosis (Chai et al. 2018; Scalvenzi et al. 2021; Shi et al. 2023; Tong, Cui, et al. 2020). However, the dynamics of Proteobacteria vary across amphibian species. Planctomycetes, though widespread in aquatic environments (Kaboré et al. 2020), are rarely detected in amphibian gut microbiomes (Shi et al. 2023; Zhang et al. 2020). The high phylogenetic divergence in TH, TF, and FL microbiomes coincides with the predominance of stochastic processes. The remodeling of gut microbiomes from TH to TF is unlikely to be driven by different species pools, as the habitat remains aquatic. The intestinal microenvironment in the forelimb phase may selectively filter microorganisms that are unsuitable for future terrestrial feeding. Temporal transmission contributes most during the final phase of metamorphosis, suggesting ecological niches remain relatively stable as fore‐limbed tadpoles develop into froglets. Comparisons of co‐occurrence networks indicate that microbial interactions during metamorphosis differ markedly from other periods. Actinobacteria and Planctomycetes probably have critical functions in network maintenance during the hindlimb stage, whereas Firmicutes become functionally dominant in later stages.

After metamorphosis froglets actively feed on land and grow rapidly. The gastrointestinal tract differentiates structurally and functionally: the slender, coiled SG is connected via mesentery, whereas the stubby, straight LG connects to the cloaca. Differences in internal environments (e.g., microvilli length and oxygen content) create distinct ecological niches for microbial colonization (Martinez‐Guryn et al. 2019). Higher oxygen in SG favors Proteobacteria colonization, while oxygen consumption by Proteobacteria may create hypoxic conditions conducive to Firmicutes and Bacteroidetes in LG. Although stochastic processes (drift) predominantly shape both SG and LG microbiomes, deterministic processes (homogeneous selection, habitat filtering, and microbial interactions) are more influential in LG. Temporal transmission contributes far more than spatial dispersal to SG microbiomes, likely due to unidirectional intestinal flow, while spatial dispersal contributes more to LG microbiomes in July, reflecting incompletely differentiated intestines at the subadult stage (Gao et al. 2018). Notably, gut microbiomes in August exhibit maximal structural differences between tissues, weakest homogeneous selection, and least robust co‐occurrence network, possibly due to decreased food intake under high temperatures and attenuated intestine function (Fontaine et al. 2018; Lian et al. 2020).

Adult amphibians typically enter hibernation to survive prolonged fasting and cold exposure, accompanied by dramatic gastrointestinal restructuring to optimize energy allocation (Naya et al. 2009). Gut microbiomes also undergo significant remodeling (Cao et al. 2023; Shi et al. 2023; Tong, Cui, et al. 2020; Tong et al. 2023; Tong, Hu, et al. 2020; Weng et al. 2016). The remodeling patterns vary highly among species. For instance, alpha diversity decreases in hibernating *Rana amurensis* , *Rana dybowskii* , *Polypedates megacephalus* , and *Strauchbufo raddei* , whereas *Odorrana Tormota* exhibits decreased species richness but initially increased evenness. In this study, species richness and phylogenetic diversity increase during pro‐ and mid‐hibernation, regardless of tissue. SG microbiomes maintain low evenness, whereas the evenness of LG microbiomes declines gradually. Proteobacteria consistently dominates SG microbiomes, while Proteobacteria and Planctomycetes increase and Firmicutes and Fusobacteria decline in LG microbiomes. Gut symbionts act as an essential regulator in material and energy metabolism during hibernation (Kurtz et al. 2021), with long‐term fasting favoring microbes capable of metabolizing endogenous substances such as mucosal proteins, apoptotic cells, and urea. For example, *Pseudomonas* in LG microbiomes may enhance urea nitrogen recycling (Regan et al. 2022; Wiebler et al. 2018). Enhanced stochasticity during hibernation also contributes to greater species richness, lower evenness, and increased rare taxa. Increased similarity between SG and LG microbiomes during hibernation likely reflects stronger homogeneous selection and reduced dispersal limitation, consistent with increased SG‐to‐LG dispersal in January.

## 5 Conclusions

This study demonstrates that amphibian gut microbiomes exhibit tissue‐specific structural succession, with temporal fluctuations in structural discrepancy between SG and LG microbiomes. Stochastic processes dominate the gut microbiome assembly, although deterministic processes—including habitat filtering and microbial interactions—remain significant, particularly for LG microbiomes. Comparisons between sexes, performed only in July and September, revealed minimal sex effects; however, gonadal differentiation likely occurs in the hindlimb stage of metamorphosis (Li et al. 2018), so the finding should be interpreted cautiously. To address small sample size, co‐occurrence networks were constructed by merging groups across tissues and months, potentially limiting detection of tissue‐ or month‐specific microbial interactions. Additionally, gut microbiomes in later and after hibernation were not profiled, though they likely restore prehibernation structure (Park and Do 2024). Further research is warranted to characterize the spatiotemporal dynamics of amphibian gut microbiomes during and after hibernation.

## Author Contributions

**Xiaowei Song:** conceptualization (lead), data curation (lead), formal analysis (lead), funding acquisition (lead), investigation (lead), methodology (lead), project administration (lead), writing – original draft (lead), writing – review and editing (lead). **Yuanyuan Zhai:** investigation (equal). **Mengyang Zhang:** investigation (equal). **Jingyuan Guo:** investigation (equal). **Benjun Guo:** data curation (equal), software (equal). **Chaolong Zhang:** data curation (equal), software (equal). **Jin Jin:** data curation (equal), software (equal). **Weiye Wang:** data curation (equal), software (equal). **Yuanping Xu:** data curation (equal), software (equal). **Bicheng Zhu:** writing – original draft (equal), writing – review and editing (equal). **Xiangzhen Li:** conceptualization (equal), writing – original draft (equal), writing – review and editing (equal).

## Funding

The study was supported by the National Natural Science Foundation of China (NSFC 31600104), Key R&D and Promotion Projects of Henan Province (192102110005), and Research Startup Foundation in Chengdu University of Information Technology (KYTZ202279).

## Ethics Statement

The animal cultivation complied with the local aquaculture regulations, and the procedures used in the research were approved by the Animal Care and Use Committee in Xinyang Normal University (XFEC‐2018‐09).

## Conflicts of Interest

The authors declare no conflicts of interest.

## Supporting information


**Data S1:** ece373041‐sup‐0001‐Supinfo1.docx.

**Table S4:** ece373041‐sup‐0002‐TableS4.tsv.

**Table S8:** ece373041‐sup‐0003‐TableS8.xlsx.

## Data Availability

The 16S metagenomic data, metadata, and R codes have been deposited in National Microbiology Data Center with accession numbers NMDC10020041 and NMDCX0002204 (https://nmdc.cn/resource/genomics/project/detail/NMDC10020041; https://nmdc.cn/resource/attachment/detail/NMDCX0002204).

## References

[ece373041-bib-0001] Amir, A. , D. McDonald , J. A. Navas‐Molina , et al. 2017. “Deblur Rapidly Resolves Single‐Nucleotide Community Sequence Patterns.” mSystems 2, no. 2: e00191‐16. 10.1128/mSystems.00191-16. 28289731 PMC5340863

[ece373041-bib-0002] Bache, S. M. , and H. Wickham . 2022. “magrittr: A Forward‐Pipe Operator for R (Version 2.0.3) [Computer Software].” https://CRAN.R‐project.org/package=magrittr.

[ece373041-bib-0003] Barrett, T. , M. Dowle , A. Srinivasan , J. Gorecki , M. Chirico , and T. Hocking . 2024. “data.table: Extension of ‘data.frame’ (Version 1.15.4) [Computer Software].” https://CRAN.R‐project.org/package=data.table.

[ece373041-bib-0004] Berg, G. , D. Rybakova , D. Fischer , et al. 2020. “Microbiome Definition Re‐Visited: Old Concepts and New Challenges.” Microbiome 8, no. 1: 103. 10.1186/s40168-020-00875-0. 32605663 PMC7329523

[ece373041-bib-0005] Bolyen, E. , J. R. Rideout , M. R. Dillon , et al. 2019. “Reproducible, Interactive, Scalable and Extensible Microbiome Data Science Using QIIME 2.” Nature Biotechnology 37, no. 8: 852–857. 10.1038/s41587-019-0209-9. PMC7015180 31341288

[ece373041-bib-0006] Brown, C. C. , and A. Y. Rudensky . 2023. “Spatiotemporal Regulation of Peripheral T Cell Tolerance.” Science 380, no. 6644: 472–478. 10.1126/science.adg6425. 37141369

[ece373041-bib-0007] Cao, H. , Y. Shi , J. Wang , et al. 2023. “The Intestinal Microbiota and Metabolic Profiles of *Strauchbufo raddei* Underwent Adaptive Changes During Hibernation.” Integrative Zoology 19: 1749‐4877.12749. 10.1111/1749-4877.12749. 37430430

[ece373041-bib-0008] Chai, L. , Z. Dong , A. Chen , and H. Wang . 2018. “Changes in Intestinal Microbiota of Bufo Gargarizans and Its Association With Body Weight During Metamorphosis.” Archives of Microbiology 200, no. 7: 1087–1099. 10.1007/s00203-018-1523-1. 29748695

[ece373041-bib-0009] Emerson, K. J. , and S. K. Woodley . 2024. “Something in the Water: Aquatic Microbial Communities Influence the Larval Amphibian Gut Microbiota, Neurodevelopment and Behaviour.” Proceedings of the Royal Society B: Biological Sciences 291, no. 2017: 20232850. 10.1098/rspb.2023.2850. PMC10898966 38412968

[ece373041-bib-0010] Fan, C. , L. Zhang , S. Jia , et al. 2022. “Seasonal Variations in the Composition and Functional Profiles of Gut Microbiota Reflect Dietary Changes in Plateau Pikas.” Integrative Zoology 17, no. 3: 379–395. 10.1111/1749-4877.12630. 35051309 PMC9305894

[ece373041-bib-0011] Fluch, M. , E. Corretto , H. Feldhaar , and H. Schuler . 2024. “Seasonal Changes in the Gut Microbiota of *Halyomorpha halys* .” Microbial Ecology 87, no. 1: 164. 10.1007/s00248-024-02481-1. 39731630 PMC11682014

[ece373041-bib-0012] Fontaine, S. S. , A. J. Novarro , and K. D. Kohl . 2018. “Environmental Temperature Alters the Digestive Performance and Gut Microbiota of a Terrestrial Amphibian.” Journal of Experimental Biology: jeb.187559. 10.1242/jeb.187559. 30171093

[ece373041-bib-0013] Gao, S. , L. Yan , R. Wang , et al. 2018. “Tracing the Temporal‐Spatial Transcriptome Landscapes of the Human Fetal Digestive Tract Using Single‐Cell RNA‐Sequencing.” Nature Cell Biology 20, no. 6: 721–734. 10.1038/s41556-018-0105-4. 29802404

[ece373041-bib-0014] Hendrycks, W. , N. Mullens , J. Bakengesa , et al. 2025. “Deterministic and Stochastic Effects Drive the Gut Microbial Diversity in Cucurbit‐Feeding Fruit Flies (Diptera, Tephritidae).” PLoS One 20, no. 1: e0313447. 10.1371/journal.pone.0313447. 39854335 PMC11759365

[ece373041-bib-0015] Hourdry, J. , A. L'Hermite , and R. Ferrand . 1996. “Changes in the Digestive Tract and Feeding Behavior of Anuran Amphibians During Metamorphosis.” Physiological Zoology 69, no. 2: 219–251. 10.1086/physzool.69.2.30164181.

[ece373041-bib-0016] Huang, C. , and W. Liao . 2021. “Seasonal Variation in Gut Microbiota Related to Diet in *Fejervarya limnocharis* .” Animals 11, no. 5: 1393. 10.3390/ani11051393. 34068415 PMC8153623

[ece373041-bib-0017] Ishizuya‐Oka, A. 2007. “Regeneration of the Amphibian Intestinal Epithelium Under the Control of Stem Cell Niche.” Development, Growth & Differentiation 49, no. 2: 99–107. 10.1111/j.1440-169X.2007.00913.x. 17335431

[ece373041-bib-0018] Kaboré, O. D. , S. Godreuil , and M. Drancourt . 2020. “Planctomycetes as Host‐Associated Bacteria: A Perspective That Holds Promise for Their Future Isolations, by Mimicking Their Native Environmental Niches in Clinical Microbiology Laboratories.” Frontiers in Cellular and Infection Microbiology 10: 519301. 10.3389/fcimb.2020.519301. 33330115 PMC7734314

[ece373041-bib-0019] Kassambara, A. 2023. “ggpubr: “ggplot2” Based Publication Ready Plots (Version 0.6.0) [Computer Software].” https://CRAN.R‐project.org/package=ggpubr.

[ece373041-bib-0020] Katoh, K. , and D. M. Standley . 2013. “MAFFT Multiple Sequence Alignment Software Version 7: Improvements in Performance and Usability.” Molecular Biology and Evolution 30, no. 4: 772–780. 10.1093/molbev/mst010. 23329690 PMC3603318

[ece373041-bib-0021] Kern, L. , S. K. Abdeen , A. A. Kolodziejczyk , and E. Elinav . 2021. “Commensal Inter‐Bacterial Interactions Shaping the Microbiota.” Current Opinion in Microbiology 63: 158–171. 10.1016/j.mib.2021.07.011. 34365152

[ece373041-bib-0022] Knutie, S. A. , C. L. Wilkinson , K. D. Kohl , and J. R. Rohr . 2017. “Early‐Life Disruption of Amphibian Microbiota Decreases Later‐Life Resistance to Parasites.” Nature Communications 8, no. 1: 86. 10.1038/s41467-017-00119-0. PMC5519754 28729558

[ece373041-bib-0023] Kohl, K. D. , T. L. Cary , W. H. Karasov , and M. D. Dearing . 2013. “Restructuring of the Amphibian Gut Microbiota Through Metamorphosis.” Environmental Microbiology Reports 5, no. 6: 899–903. 10.1111/1758-2229.12092. 24249298

[ece373041-bib-0024] Kurtz, C. C. , J. P. Otis , M. D. Regan , and H. V. Carey . 2021. “How the Gut and Liver Hibernate.” Comparative Biochemistry and Physiology Part A: Molecular & Integrative Physiology 253: 110875. 10.1016/j.cbpa.2020.110875. PMC7867651 33348019

[ece373041-bib-0025] Legendre, P. 2008. “Studying Beta Diversity: Ecological Variation Partitioning by Multiple Regression and Canonical Analysis.” Journal of Plant Ecology 1, no. 1: 3–8. 10.1093/jpe/rtm001.

[ece373041-bib-0026] Levin, D. , N. Raab , Y. Pinto , et al. 2021. “Diversity and Functional Landscapes in the Microbiota of Animals in the Wild.” Science 372, no. 6539: eabb5352. 10.1126/science.abb5352. 33766942

[ece373041-bib-0027] Li, Y.‐Y. , T. Meng , K. Gao , and Z.‐F. Qin . 2018. “Gonadal Differentiation and Its Sensitivity to Androgens During Development of *Pelophylax nigromaculatus* .” Aquatic Toxicology 202: 188–195. 10.1016/j.aquatox.2018.07.016. 30056249

[ece373041-bib-0028] Lian, P. , S. Braber , J. Garssen , et al. 2020. “Beyond Heat Stress: Intestinal Integrity Disruption and Mechanism‐Based Intervention Strategies.” Nutrients 12, no. 3: 734. 10.3390/nu12030734. 32168808 PMC7146479

[ece373041-bib-0029] Liu, C. , Y. Cui , X. Li , and M. Yao . 2021. “ *Microeco*: An R Package for Data Mining in Microbial Community Ecology.” FEMS Microbiology Ecology 97, no. 2: fiaa255. 10.1093/femsec/fiaa255. 33332530

[ece373041-bib-0030] Liu, C. , C. Li , Y. Jiang , R. J. Zeng , M. Yao , and X. Li . 2023. “A Guide for Comparing Microbial Co‐Occurrence Networks.” iMeta 2, no. 1: e71. 10.1002/imt2.71. 38868345 PMC10989802

[ece373041-bib-0031] Lu, H.‐P. , Y.‐C. Yeh , A. R. Sastri , F.‐K. Shiah , G.‐C. Gong , and C. Hsieh . 2016. “Evaluating Community–Environment Relationships Along Fine to Broad Taxonomic Resolutions Reveals Evolutionary Forces Underlying Community Assembly.” ISME Journal 10, no. 12: 2867–2878. 10.1038/ismej.2016.78. 27177191 PMC5148199

[ece373041-bib-0032] Martinez‐Guryn, K. , V. Leone , and E. B. Chang . 2019. “Regional Diversity of the Gastrointestinal Microbiome.” Cell Host & Microbe 26, no. 3: 314–324. 10.1016/j.chom.2019.08.011. 31513770 PMC6750279

[ece373041-bib-0033] Naya, D. E. , C. Veloso , P. Sabat , and F. Bozinovic . 2009. “The Effect of Short‐ and Long‐Term Fasting on Digestive and Metabolic Flexibility in the Andean Toad, *Bufo spinulosus* .” Journal of Experimental Biology 212, no. 14: 2167–2175. 10.1242/jeb.030650. 19561206

[ece373041-bib-0034] Ning, D. , M. Yuan , L. Wu , et al. 2020. “A Quantitative Framework Reveals Ecological Drivers of Grassland Microbial Community Assembly in Response to Warming.” Nature Communications 11, no. 1: 4717. 10.1038/s41467-020-18560-z. PMC7501310 32948774

[ece373041-bib-0035] Oksanen, J. , G. L. Simpson , F. G. Blanchet , et al. 2022. “vegan: Community Ecology Package (Version 2.6‐4) [Computer Software].” https://CRAN.R‐project.org/package=vegan.

[ece373041-bib-0036] Paradis, E. , and K. Schliep . 2019. “Ape 5.0: An Environment for Modern Phylogenetics and Evolutionary Analyses in R.” Bioinformatics 35, no. 3: 526–528. 10.1093/bioinformatics/bty633. 30016406

[ece373041-bib-0037] Park, J. , and Y. Do . 2024. “The Difference and Variation of Gut Bacterial Community and Host Physiology Can Support Adaptation During and After Overwintering in Frog Population.” Integrative Zoology 19, no. 4. 10.1111/1749-4877.12798. 38185804

[ece373041-bib-0038] Price, M. N. , P. S. Dehal , and A. P. Arkin . 2010. “FastTree 2—Approximately Maximum‐Likelihood Trees for Large Alignments.” PLoS One 5, no. 3: e9490. 10.1371/journal.pone.0009490. 20224823 PMC2835736

[ece373041-bib-0039] Quast, C. , E. Pruesse , P. Yilmaz , et al. 2012. “The SILVA Ribosomal RNA Gene Database Project: Improved Data Processing and Web‐Based Tools.” Nucleic Acids Research 41, no. D1: D590–D596. 10.1093/nar/gks1219. 23193283 PMC3531112

[ece373041-bib-0040] R Core Team . 2024. R: A Language and Environment for Statistical Computing [Computer Software]. R Foundation for Statistical Computing. https://www.R‐project.org.

[ece373041-bib-0041] Regan, M. D. , E. Chiang , Y. Liu , et al. 2022. “Nitrogen Recycling via Gut Symbionts Increases in Ground Squirrels Over the Hibernation Season.” Science 375, no. 6579: 460–463. 10.1126/science.abh2950. 35084962 PMC8936132

[ece373041-bib-0042] Scalvenzi, T. , I. Clavereau , M. Bourge , and N. Pollet . 2021. “Gut Microbial Ecology of Xenopus Tadpoles Across Life Stages.” Peer Community Journal 1: e41. 10.24072/pcjournal.53.

[ece373041-bib-0043] Segata, N. , J. Izard , L. Waldron , et al. 2011. “Metagenomic Biomarker Discovery and Explanation.” Genome Biology 12, no. 6: R60. 10.1186/gb-2011-12-6-r60. 21702898 PMC3218848

[ece373041-bib-0044] Shade, A. , J. Gregory Caporaso , J. Handelsman , R. Knight , and N. Fierer . 2013. “A Meta‐Analysis of Changes in Bacterial and Archaeal Communities With Time.” ISME Journal 7, no. 8: 1493–1506. 10.1038/ismej.2013.54. 23575374 PMC3721121

[ece373041-bib-0045] Shenhav, L. , M. Thompson , T. A. Joseph , et al. 2019. “FEAST: Fast Expectation‐Maximization for Microbial Source Tracking.” Nature Methods 16, no. 7: 627–632. 10.1038/s41592-019-0431-x. 31182859 PMC8535041

[ece373041-bib-0046] Shi, Q. , Y. Li , S. Deng , et al. 2023. “The Succession of Gut Microbiota in the Concave‐Eared Torrent Frog (*Odorrana tormota*) Throughout Developmental History.” Ecology and Evolution 13, no. 5: e10094. 10.1002/ece3.10094. 37214611 PMC10199338

[ece373041-bib-0047] Song, X. , J. Song , H. Song , Q. Zeng , and K. Shi . 2018. “A Robust Noninvasive Approach to Study Gut Microbiota Structure of Amphibian Tadpoles by Feces.” Asian Herpetological Research 9, no. 1: 1–12. 10.16373/j.cnki.ahr.170062.

[ece373041-bib-0048] Song, X. , Y. Zhai , J. Song , J. Zhang , and X. Li . 2023. “The Structural Discrepancy Between the Small and Large Gut Microbiota of Asiatic Toad ( *Bufo gargarizans* ) During Hibernation.” Folia Microbiologica 68, no. 4: 537–546. 10.1007/s12223-023-01031-5. 36637770

[ece373041-bib-0049] Song, X. , J. Zhang , J. Song , and Y. Zhai . 2021. “Decisive Effects of Life Stage on the Gut Microbiota Discrepancy Between Two Wild Populations of Hibernating Asiatic Toads ( *Bufo gargarizans* ).” Frontiers in Microbiology 12: 665849. 10.3389/fmicb.2021.665849. 34413833 PMC8369469

[ece373041-bib-0050] Tong, Q. , L.‐Y. Cui , Z.‐F. Hu , X.‐P. Du , H. M. Abid , and H.‐B. Wang . 2020. “Environmental and Host Factors Shaping the Gut Microbiota Diversity of Brown Frog *Rana Dybowskii* .” Science of the Total Environment 741: 140142. 10.1016/j.scitotenv.2020.140142. 32615421

[ece373041-bib-0051] Tong, Q. , W. Dong , M. Xu , et al. 2023. “Characteristics and a Comparison of the Gut Microbiota in Two Frog Species at the Beginning and End of Hibernation.” Frontiers in Microbiology 14: 1057398. 10.3389/fmicb.2023.1057398. 37206336 PMC10191234

[ece373041-bib-0052] Tong, Q. , Z. Hu , X. Du , J. Bie , and H. Wang . 2020. “Effects of Seasonal Hibernation on the Similarities Between the Skin Microbiota and Gut Microbiota of an Amphibian (*Rana dybowskii*).” Microbial Ecology 79, no. 4: 898–909. 10.1007/s00248-019-01466-9. 31820074

[ece373041-bib-0053] Tran, M. , J. R. Huh , and A. S. Devlin . 2025. “The Role of Gut Microbial Metabolites in the T Cell Lifecycle.” Nature Immunology 26, no. 8: 1246–1257. 10.1038/s41590-025-02227-2. 40691327 PMC13124069

[ece373041-bib-0054] Wang, Z. , Y. Wang , Z. He , et al. 2025. “Research Status and Prospect of Amphibian Symbiotic Microbiota.” Animals 15, no. 7: 934. 10.3390/ani15070934. 40218328 PMC11987896

[ece373041-bib-0055] Warne, R. W. , L. Kirschman , and L. Zeglin . 2019. “Manipulation of Gut Microbiota During Critical Developmental Windows Affects Host Physiological Performance and Disease Susceptibility Across Ontogeny.” Journal of Animal Ecology 88, no. 6: 845–856. 10.1111/1365-2656.12973. 30828805

[ece373041-bib-0056] Weng, F. C.‐H. , Y.‐J. Yang , and D. Wang . 2016. “Functional Analysis for Gut Microbes of the Brown Tree Frog (*Polypedates megacephalus*) in Artificial Hibernation.” BMC Genomics 17, no. S13: 1024. 10.1186/s12864-016-3318-6. 28155661 PMC5260014

[ece373041-bib-0057] Wiebler, J. M. , K. D. Kohl , R. E. Lee , and J. P. Costanzo . 2018. “Urea Hydrolysis by Gut Bacteria in a Hibernating Frog: Evidence for Urea‐Nitrogen Recycling in Amphibia.” Proceedings of the Royal Society B: Biological Sciences 285, no. 1878: 20180241. 10.1098/rspb.2018.0241. PMC5966601 29720413

[ece373041-bib-0058] Xu, L. , P. Xiang , X. Liu , et al. 2024. “Deterministic Processes Dominate Microbial Assembly Mechanisms in the Gut Microbiota of Cold‐Water Fish Between Summer and Winter.” Frontiers in Microbiology 15: 1415931. 10.3389/fmicb.2024.1415931. 38952450 PMC11216611

[ece373041-bib-0059] Zhang, M. , H. Chen , L. Liu , et al. 2020. “The Changes in the Frog Gut Microbiome and Its Putative Oxygen‐Related Phenotypes Accompanying the Development of Gastrointestinal Complexity and Dietary Shift.” Frontiers in Microbiology 11: 162. 10.3389/fmicb.2020.00162. 32194513 PMC7062639

[ece373041-bib-0060] Zhang, M. , S. Gaughan , Q. Chang , et al. 2019. “Age‐Related Changes in the Gut Microbiota of the Chinese Giant Salamander (*Andrias davidianus*).” MicrobiologyOpen 8, no. 7: e00778. 10.1002/mbo3.778. 30585426 PMC6612560

[ece373041-bib-0061] Zhou, J. , and D. Ning . 2017. “Stochastic Community Assembly: Does It Matter in Microbial Ecology?” Microbiology and Molecular Biology Reviews 81, no. 4: e00002‐17. 10.1128/MMBR.00002-17. 29021219 PMC5706748

[ece373041-bib-0062] Zhu, B. , C. Shao , W. Xu , J. Dai , G. Fu , and Y. Hu . 2024. “Effects of Thyroid Powder on Tadpole (*Lithobates catesbeiana*) Metamorphosis and Growth: The Role of Lipid Metabolism and Gut Microbiota.” Animals 14, no. 2: 208. 10.3390/ani14020208. 38254377 PMC10812769

[ece373041-bib-0063] Zilber‐Rosenberg, I. , and E. Rosenberg . 2008. “Role of Microorganisms in the Evolution of Animals and Plants: The Hologenome Theory of Evolution.” FEMS Microbiology Reviews 32, no. 5: 723–735. 10.1111/j.1574-6976.2008.00123.x. 18549407

