# Supplementary material for: Stochasticity Prevails but Differs: Tissue‐Specific Assembly of Gut Microbiomes Across Seasons in an Amphibian Model
Source: Ecol Evol. 2026 Feb 3;16(2):e73041. doi: 10.1002/ece3.73041 (PMC12867956; doi:10.1002/ece3.73041)
Supplement: Supplementary file 1 — Data S1: ece373041‐sup‐0001‐Supinfo1.docx. [file ECE3-16-e73041-s003.docx]

**Supplementary Fig 1 Principal coordinates analysis (PCoA) on the four beta diversity indices of 108 gut microbiomes (17 sample groups). The scale value in square brackets next to the coordinate represents the variance explained by the coordinate.**


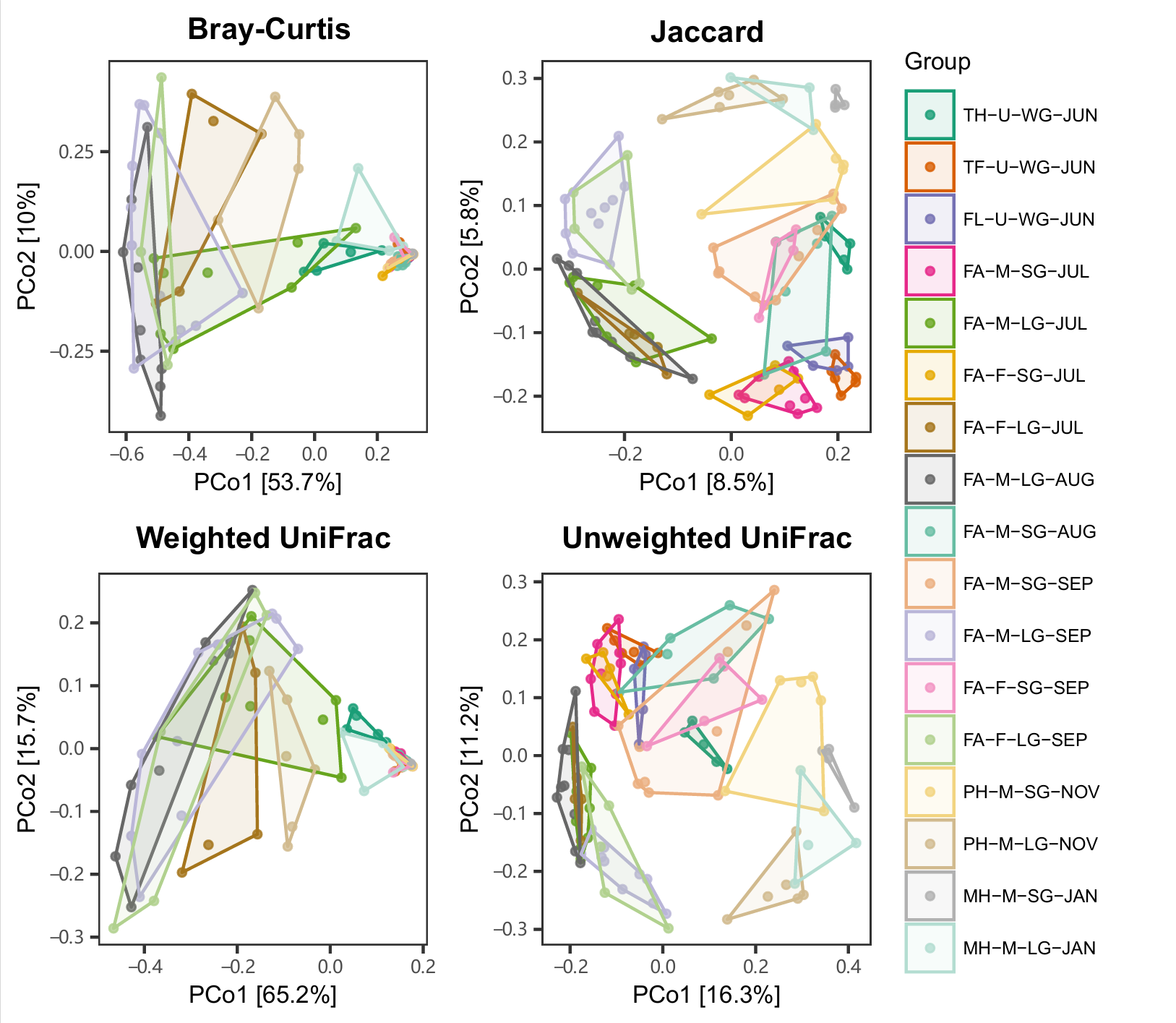


**Supplementary Fig 2 The temporal dynamics of SG (a) and LG (b) microbiomes in terms of Phylum, Family, and Genus levels. WG microbiomes were integrated in the analyses on SG and LG microbiomes, but female samples were excluded.** **The taxa were merged into Others except for the top 10 taxa in mean relative abundance.**


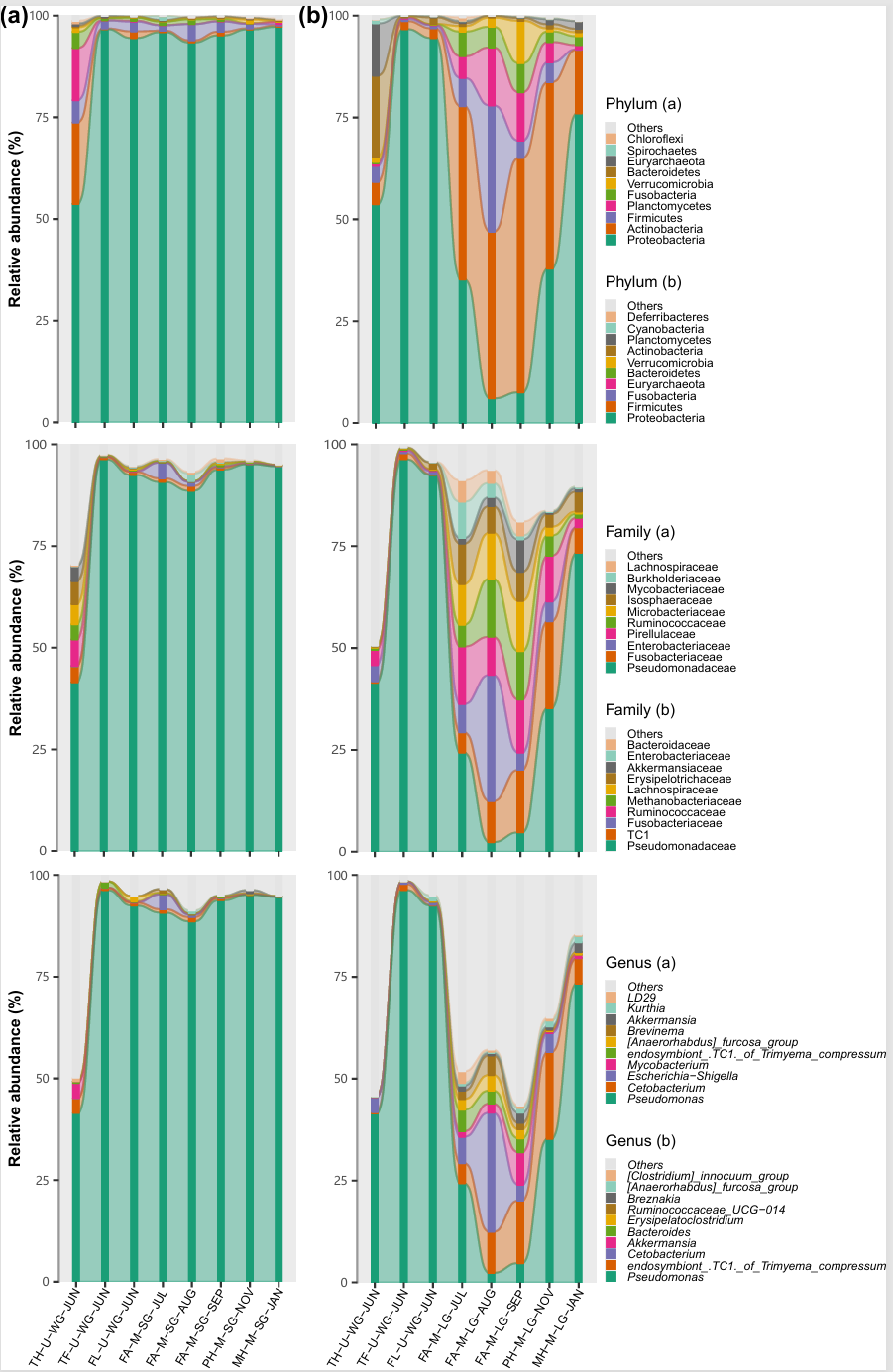


**Supplementary Fig 3 The temporal dynamics of pairwise shared-ASV ratios and beta diversity indices between SG and LG microbiomes (i.e., only male samples from July, August, September, November and January). The significance level for Wilcoxon rank sum tests was set to 0.05.**


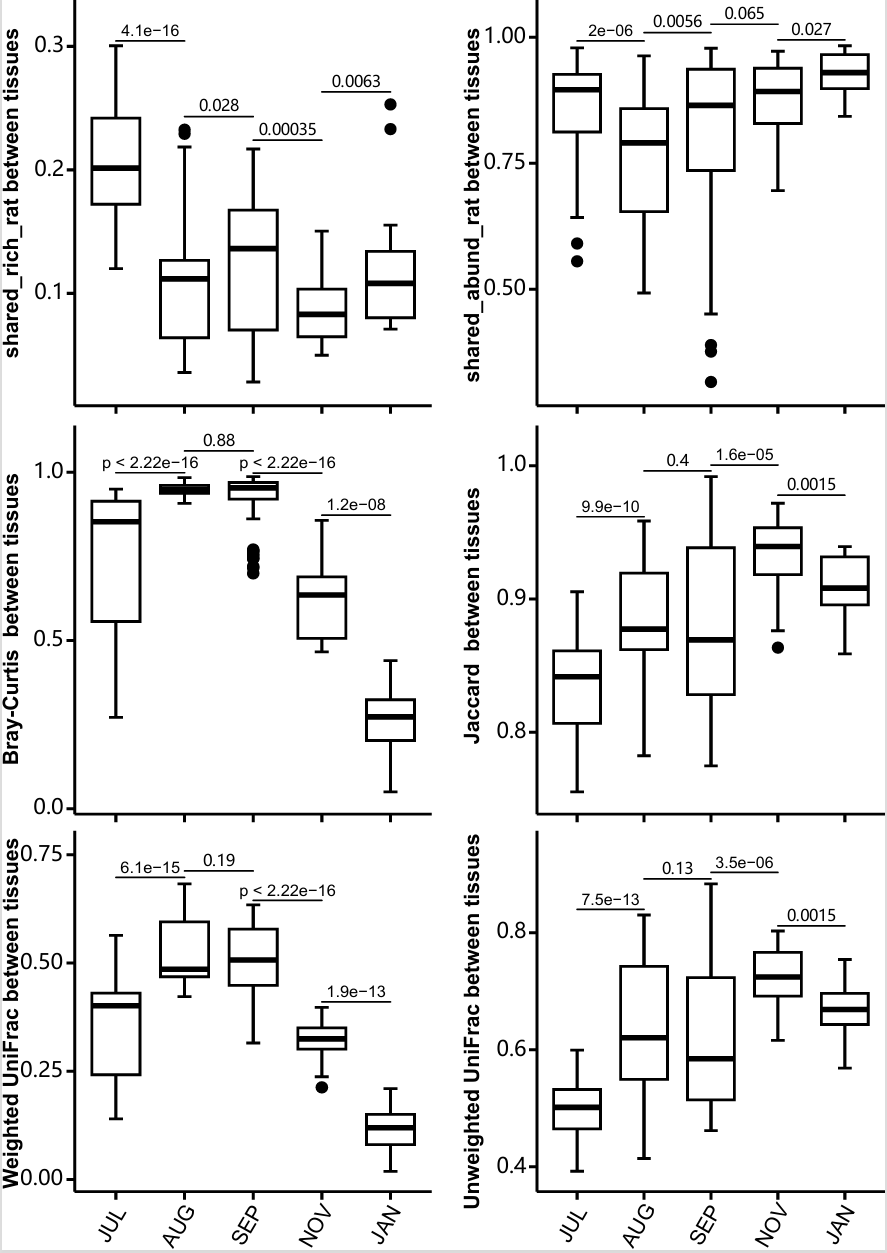


**Supplementary Fig 4 The robustness measures for co-occurrence networks of eight gut microbiome groups. The Eff and Eigen represent two robustness measures (i.e., network efficiency and natural connectivity), respectively. The edge_rand, edge_strong, node_rand and node_degree_high represent four removal strategies. † M_SG: male small gut microbiomes; M_LG: male large gut microbiomes; WG: whole gut microbiomes in June; M_JUL: male gut microbiomes in July; M_AUG: male gut microbiomes in August; M_SEP: male gut microbiomes in September; M_NOV: male gut microbiomes in November; M_JAN: male gut microbiomes in January.**


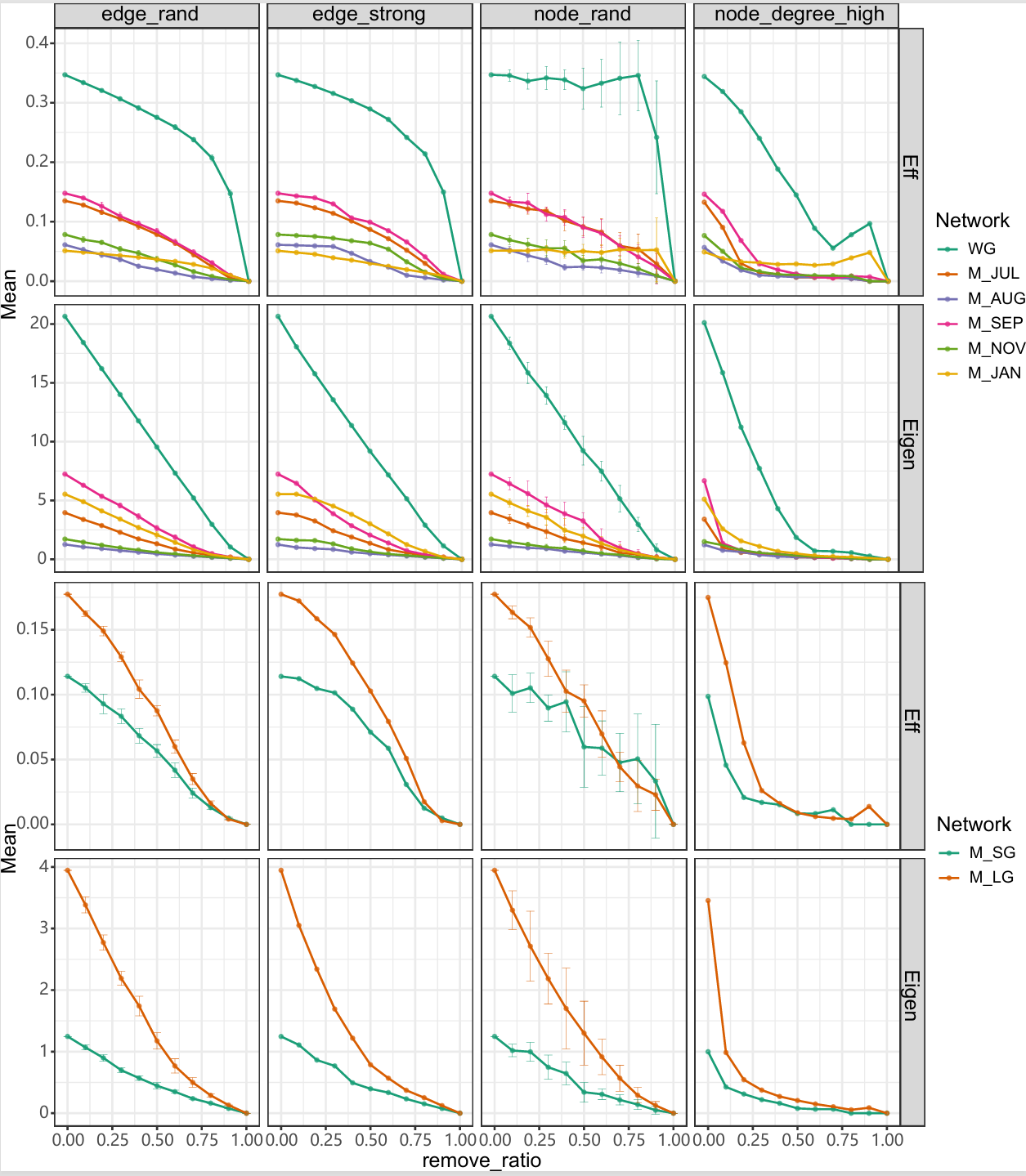


Supplementary Table 1 The metadata of 110 gut microbiomes (17 sample groups) in 65 frog individuals under rice-frog co-cultivation mode.

| **Microbiome ID** | **Frog ID** | **Life stage** | **Sex** | **Tissue** | **Month** | **Life span (dpf)** | **SVL (mm)** | **Temperature (℃)** | **Group** |
| --- | --- | --- | --- | --- | --- | --- | --- | --- | --- |
| IA76 | IA76 | TH | U | WG | JUN | 57 | 20.70 | 25.92 | TH-U-WG-JUN |
| IA77 | IA77 | TH | U | WG | JUN | 57 | 19.20 | 25.92 | TH-U-WG-JUN |
| IA81 | IA81 | TH | U | WG | JUN | 57 | 22.60 | 25.92 | TH-U-WG-JUN |
| IA82 | IA82 | TH | U | WG | JUN | 57 | 20.60 | 25.92 | TH-U-WG-JUN |
| IA83 | IA83 | TH | U | WG | JUN | 57 | 19.10 | 25.92 | TH-U-WG-JUN |
| IA70 | IA70 | TF | U | WG | JUN | 57 | 22.68 | 25.92 | TF-U-WG-JUN |
| IA72 | IA72 | TF | U | WG | JUN | 57 | 22.00 | 25.92 | TF-U-WG-JUN |
| IA73 | IA73 | TF | U | WG | JUN | 57 | 23.00 | 25.92 | TF-U-WG-JUN |
| IA71 | IA71 | TF | U | WG | JUN | 57 | 22.40 | 25.92 | TF-U-WG-JUN |
| IA75 | IA75 | TF | U | WG | JUN | 57 | 23.84 | 25.92 | TF-U-WG-JUN |
| IA74 | IA74 | TF | U | WG | JUN | 57 | 21.84 | 25.92 | TF-U-WG-JUN |
| IA44 | IA44 | FL | U | WG | JUN | 57 | 24.52 | 25.92 | FL-U-WG-JUN |
| IA45 | IA45 | FL | U | WG | JUN | 57 | 27.20 | 25.92 | FL-U-WG-JUN |
| IA50 | IA50 | FL | U | WG | JUN | 57 | 23.40 | 25.92 | FL-U-WG-JUN |
| IA51 | IA51 | FL | U | WG | JUN | 57 | 25.40 | 25.92 | FL-U-WG-JUN |
| IA54 | IA54 | FL | U | WG | JUN | 57 | 22.12 | 25.92 | FL-U-WG-JUN |
| IIAS22 | IIA22 | FA | M | SG | JUL | 93 | 27.78 | 29.68 | FA-M-SG-JUL |
| IIAZ22 | IIA22 | FA | M | LG | JUL | 93 | 27.78 | 29.68 | FA-M-LG-JUL |
| IIAS38 | IIA38 | FA | M | SG | JUL | 93 | 28.48 | 29.68 | FA-M-SG-JUL |
| IIAS21 | IIA21 | FA | M | SG | JUL | 93 | 32.46 | 29.68 | FA-M-SG-JUL |
| IIAZ21 | IIA21 | FA | M | LG | JUL | 93 | 32.46 | 29.68 | FA-M-LG-JUL |
| IIAS05 | IIA05 | FA | M | SG | JUL | 93 | 34.00 | 29.68 | FA-M-SG-JUL |
| IIAZ05 | IIA05 | FA | M | LG | JUL | 93 | 34.00 | 29.68 | FA-M-LG-JUL |
| IIAS34 | IIA34 | FA | M | SG | JUL | 93 | 36.24 | 29.68 | FA-M-SG-JUL |
| IIAZ34 | IIA34 | FA | M | LG | JUL | 93 | 36.24 | 29.68 | FA-M-LG-JUL |
| IIAS04 | IIA04 | FA | M | SG | JUL | 93 | 36.38 | 29.68 | FA-M-SG-JUL |
| IIAZ04 | IIA04 | FA | M | LG | JUL | 93 | 36.38 | 29.68 | FA-M-LG-JUL |
| IIAS44 | IIA44 | FA | M | SG | JUL | 93 | 36.80 | 29.68 | FA-M-SG-JUL |
| IIAZ44 | IIA44 | FA | M | LG | JUL | 93 | 36.80 | 29.68 | FA-M-LG-JUL |
| IIAS24 | IIA24 | FA | M | SG | JUL | 93 | 41.42 | 29.68 | FA-M-SG-JUL |
| IIAZ24 | IIA24 | FA | M | LG | JUL | 93 | 41.42 | 29.68 | FA-M-LG-JUL |
| IIAS03 | IIA03 | FA | M | SG | JUL | 93 | 41.62 | 29.68 | FA-M-SG-JUL |
| IIAZ03 | IIA03 | FA | M | LG | JUL | 93 | 41.62 | 29.68 | FA-M-LG-JUL |
| IIAS47 | IIA47 | FA | F | SG | JUL | 93 | 31.24 | 29.68 | FA-F-SG-JUL |
| IIAZ47 | IIA47 | FA | F | LG | JUL | 93 | 31.24 | 29.68 | FA-F-LG-JUL |
| IIAS20 | IIA20 | FA | F | SG | JUL | 93 | 31.96 | 29.68 | FA-F-SG-JUL |
| IIAZ20 | IIA20 | FA | F | LG | JUL | 93 | 31.96 | 29.68 | FA-F-LG-JUL |
| IIAS35 | IIA35 | FA | F | SG | JUL | 93 | 34.16 | 29.68 | FA-F-SG-JUL |
| IIAZ35 | IIA35 | FA | F | LG | JUL | 93 | 34.16 | 29.68 | FA-F-LG-JUL |
| IIAS27 | IIA27 | FA | F | SG | JUL | 93 | 35.44 | 29.68 | FA-F-SG-JUL |
| IIAZ27 | IIA27 | FA | F | LG | JUL | 93 | 35.44 | 29.68 | FA-F-LG-JUL |
| IIAS26 | IIA26 | FA | F | SG | JUL | 93 | 37.00 | 29.68 | FA-F-SG-JUL |
| IIAZ26 | IIA26 | FA | F | LG | JUL | 93 | 37.00 | 29.68 | FA-F-LG-JUL |
| IIIAS30 | IIIA30 | FA | M | SG | AUG | 141 | 61.10 | 28.63 | FA-M-SG-AUG |
| IIIAZ30 | IIIA30 | FA | M | LG | AUG | 141 | 61.10 | 28.63 | FA-M-LG-AUG |
| IIIAZ12 | IIIA12 | FA | M | LG | AUG | 141 | 61.60 | 28.63 | FA-M-LG-AUG |
| IIIAS25 | IIIA25 | FA | M | SG | AUG | 141 | 64.00 | 28.63 | FA-M-SG-AUG |
| IIIAZ25 | IIIA25 | FA | M | LG | AUG | 141 | 64.00 | 28.63 | FA-M-LG-AUG |
| IIIAS51 | IIIA51 | FA | M | SG | AUG | 141 | 64.14 | 28.63 | FA-M-SG-AUG |
| IIIAZ51 | IIIA51 | FA | M | LG | AUG | 141 | 64.14 | 28.63 | FA-M-LG-AUG |
| IIIAS32 | IIIA32 | FA | M | SG | AUG | 141 | 65.00 | 28.63 | FA-M-SG-AUG |
| IIIAZ32 | IIIA32 | FA | M | LG | AUG | 141 | 65.00 | 28.63 | FA-M-LG-AUG |
| IIIAS28 | IIIA28 | FA | M | SG | AUG | 141 | 65.26 | 28.63 | FA-M-SG-AUG |
| IIIAZ28 | IIIA28 | FA | M | LG | AUG | 141 | 65.26 | 28.63 | FA-M-LG-AUG |
| IIIAS07 | IIIA07 | FA | M | SG | AUG | 141 | 64.78 | 28.63 | FA-M-SG-AUG |
| IIIAZ07 | IIIA07 | FA | M | LG | AUG | 141 | 64.78 | 28.63 | FA-M-LG-AUG |
| IIIAS43 | IIIA43 | FA | M | SG | AUG | 141 | 66.64 | 28.63 | FA-M-SG-AUG |
| IIIAZ43 | IIIA43 | FA | M | LG | AUG | 141 | 66.64 | 28.63 | FA-M-LG-AUG |
| IIIAS19 | IIIA19 | FA | M | SG | AUG | 141 | 66.70 | 28.63 | FA-M-SG-AUG |
| IIIAZ19 | IIIA19 | FA | M | LG | AUG | 141 | 66.70 | 28.63 | FA-M-LG-AUG |
| IVAS36 | IVA36 | FA | M | SG | SEP | 175 | 63.60 | 22.73 | FA-M-SG-SEP |
| IVAZ36 | IVA36 | FA | M | LG | SEP | 175 | 63.60 | 22.73 | FA-M-LG-SEP |
| IVAS44 | IVA44 | FA | M | SG | SEP | 175 | 63.78 | 22.73 | FA-M-SG-SEP |
| IVAZ44 | IVA44 | FA | M | LG | SEP | 175 | 63.78 | 22.73 | FA-M-LG-SEP |
| IVAS02 | IVA02 | FA | M | SG | SEP | 175 | 66.26 | 22.73 | FA-M-SG-SEP |
| IVAZ02 | IVA02 | FA | M | LG | SEP | 175 | 66.26 | 22.73 | FA-M-LG-SEP |
| IVAS38 | IVA38 | FA | M | SG | SEP | 175 | 66.30 | 22.73 | FA-M-SG-SEP |
| IVAZ38 | IVA38 | FA | M | LG | SEP | 175 | 66.30 | 22.73 | FA-M-LG-SEP |
| IVAS16 | IVA16 | FA | M | SG | SEP | 175 | 66.44 | 22.73 | FA-M-SG-SEP |
| IVAZ16 | IVA16 | FA | M | LG | SEP | 175 | 66.44 | 22.73 | FA-M-LG-SEP |
| IVAS19 | IVA19 | FA | M | SG | SEP | 175 | 66.50 | 22.73 | FA-M-SG-SEP |
| IVAZ19 | IVA19 | FA | M | LG | SEP | 175 | 66.50 | 22.73 | FA-M-LG-SEP |
| IVAS05 | IVA05 | FA | M | SG | SEP | 175 | 66.70 | 22.73 | FA-M-SG-SEP |
| IVAZ05 | IVA05 | FA | M | LG | SEP | 175 | 66.70 | 22.73 | FA-M-LG-SEP |
| IVAS14 | IVA14 | FA | M | SG | SEP | 175 | 67.80 | 22.73 | FA-M-SG-SEP |
| IVAZ14 | IVA14 | FA | M | LG | SEP | 175 | 67.80 | 22.73 | FA-M-LG-SEP |
| IVAS07 | IVA07 | FA | M | SG | SEP | 175 | 70.60 | 22.73 | FA-M-SG-SEP |
| IVAZ07 | IVA07 | FA | M | LG | SEP | 175 | 70.60 | 22.73 | FA-M-LG-SEP |
| IVAS29 | IVA29 | FA | M | SG | SEP | 175 | 70.90 | 22.73 | FA-M-SG-SEP |
| IVAZ29 | IVA29 | FA | M | LG | SEP | 175 | 70.90 | 22.73 | FA-M-LG-SEP |
| IVAS08 | IVA08 | FA | F | SG | SEP | 175 | 66.30 | 22.73 | FA-F-SG-SEP |
| IVAZ08 | IVA08 | FA | F | LG | SEP | 175 | 66.30 | 22.73 | FA-F-LG-SEP |
| IVAS39 | IVA39 | FA | F | SG | SEP | 175 | 67.14 | 22.73 | FA-F-SG-SEP |
| IVAS31 | IVA31 | FA | F | SG | SEP | 175 | 67.16 | 22.73 | FA-F-SG-SEP |
| IVAZ31 | IVA31 | FA | F | LG | SEP | 175 | 67.16 | 22.73 | FA-F-LG-SEP |
| IVAS10 | IVA10 | FA | F | SG | SEP | 175 | 68.70 | 22.73 | FA-F-SG-SEP |
| IVAZ10 | IVA10 | FA | F | LG | SEP | 175 | 68.70 | 22.73 | FA-F-LG-SEP |
| IVAZ47 | IVA47 | FA | F | LG | SEP | 175 | 69.42 | 22.73 | FA-F-LG-SEP |
| IVAS42 | IVA42 | FA | F | SG | SEP | 175 | 69.66 | 22.73 | FA-F-SG-SEP |
| IVAZ42 | IVA42 | FA | F | LG | SEP | 175 | 69.66 | 22.73 | FA-F-LG-SEP |
| HF1S | HF1 | PH | M | SG | NOV | 213 | 65.24 | 15.80 | PH-M-SG-NOV |
| HF1Z | HF1 | PH | M | LG | NOV | 213 | 65.24 | 15.80 | PH-M-LG-NOV |
| HF2S | HF2 | PH | M | SG | NOV | 213 | 62.82 | 15.80 | PH-M-SG-NOV |
| HF2Z | HF2 | PH | M | LG | NOV | 213 | 62.82 | 15.80 | PH-M-LG-NOV |
| HF4S | HF4 | PH | M | SG | NOV | 213 | 67.02 | 15.80 | PH-M-SG-NOV |
| HF4Z | HF4 | PH | M | LG | NOV | 213 | 67.02 | 15.80 | PH-M-LG-NOV |
| HF6S | HF6 | PH | M | SG | NOV | 213 | 72.62 | 15.80 | PH-M-SG-NOV |
| HF6Z | HF6 | PH | M | LG | NOV | 213 | 72.62 | 15.80 | PH-M-LG-NOV |
| HF7S | HF7 | PH | M | SG | NOV | 213 | 59.72 | 15.80 | PH-M-SG-NOV |
| HF7Z | HF7 | PH | M | LG | NOV | 213 | 59.72 | 15.80 | PH-M-LG-NOV |
| HF8S | HF8 | PH | M | SG | NOV | 213 | 64.78 | 15.80 | PH-M-SG-NOV |
| HF8Z | HF8 | PH | M | LG | NOV | 213 | 64.78 | 15.80 | PH-M-LG-NOV |
| HF15S | HF15 | MH | M | SG | JAN | 282 | 72.24 | 6.35 | MH-M-SG-JAN |
| HF15Z | HF15 | MH | M | LG | JAN | 282 | 72.24 | 6.35 | MH-M-LG-JAN |
| HF20S | HF20 | MH | M | SG | JAN | 282 | 59.46 | 6.35 | MH-M-SG-JAN |
| HF20Z | HF20 | MH | M | LG | JAN | 282 | 59.46 | 6.35 | MH-M-LG-JAN |
| HF21S | HF21 | MH | M | SG | JAN | 282 | 61.32 | 6.35 | MH-M-SG-JAN |
| HF21Z | HF21 | MH | M | LG | JAN | 282 | 61.32 | 6.35 | MH-M-LG-JAN |
| HF22S | HF22 | MH | M | SG | JAN | 282 | 61.02 | 6.35 | MH-M-SG-JAN |
| HF22Z | HF22 | MH | M | LG | JAN | 282 | 61.02 | 6.35 | MH-M-LG-JAN |

† The sample groups were named with the integrated format of life stage, sex, tissue, and month. TH: hind-limbed tadpole; TF: fore-limbed tadpole; FL: froglet; FA: active frog; PH: pre-hibernation; MH: mid-hibernation; F: female; M: male; U: unknown; WG: whole gut; SG: small gut; LG: large gut; dpf: days post fermentation; SVL: snout-vent length.

‡ Temperature represents the daily average temperature of rice paddy in 10 days before sampling.

**Supplementary Table 2** **The chi-square (*Χ*^2^) tests on proportions of ASV categories between temporally adjacent gut microbiomes. WG microbiomes were integrated in the analyses of SG and LG microbiomes, but female samples were excluded.**

| **ASV number** | **Group 1** | **Group 2** | ***P*(*Χ*^2^)** |
| --- | --- | --- | --- |
| **5274 (SG microbiomes)** | TH-U-WG-JUN | TF-U-WG-JUN | <0.001 |
|  | TF-U-WG-JUN | FL-U-WG-JUN | 0.140 |
|  | FL-U-WG-JUN | FA-M-SG-JUL | <0.001 |
|  | FA-M-SG-JUL | FA-M-SG-AUG | <0.001 |
|  | FA-M-SG-AUG | FA-M-SG-SEP | <0.001 |
|  | FA-M-SG-SEP | PH-M-SG-NOV | <0.001 |
|  | PH-M-SG-NOV | MH-M-SG-JAN | <0.001 |
| **6294 (LG microbiomes)** | TH-U-WG-JUN | TF-U-WG-JUN | <0.001 |
|  | TF-U-WG-JUN | FL-U-WG-JUN | 0.140 |
|  | FL-U-WG-JUN | FA-M-LG-JUL | <0.001 |
|  | FA-M-LG-JUL | FA-M-LG-AUG | <0.001 |
|  | FA-M-LG-AUG | FA-M-LG-SEP | <0.001 |
|  | FA-M-LG-SEP | PH-M-LG-NOV | <0.001 |
|  | PH-M-LG-NOV | MH-M-LG-JAN | <0.001 |

**Supplementary Table 3 The chi-square (*Χ*^2^) tests on proportions of ASV fluctuation types between temporally adjacent gut microbiomes. WG microbiomes were integrated in the analyses of SG and LG microbiomes, but female samples were excluded.**

| **ASV number** | **Group 1** | **Group 2** | ***P*(*Χ*^2^)** |
| --- | --- | --- | --- |
| **5274 (SG microbiomes)** | TH-U-WG-JUN\|TF-U-WG-JUN | TF-U-WG-JUN\|FL-U-WG-JUN | <0.001 |
|  | TF-U-WG-JUN\|FL-U-WG-JUN | FL-U-WG-JUN\|FA-M-SG-JUL | <0.001 |
|  | FL-U-WG-JUN\|FA-M-SG-JUL | FA-M-SG-JUL\|FA-M-SG-AUG | 0.638 |
|  | FA-M-SG-JUL\|FA-M-SG-AUG | FA-M-SG-AUG\|FA-M-SG-SEP | <0.001 |
|  | FA-M-SG-AUG\|FA-M-SG-SEP | FA-M-SG-SEP\|PH-M-SG-NOV | <0.001 |
|  | FA-M-SG-SEP\|PH-M-SG-NOV | PH-M-SG-NOV\|MH-M-SG-JAN | <0.001 |
| **6294 (LG microbiomes)** | TH-U-WG-JUN\|TF-U-WG-JUN | TF-U-WG-JUN\|FL-U-WG-JUN | <0.001 |
|  | TF-U-WG-JUN\|FL-U-WG-JUN | FL-U-WG-JUN\|FA-M-LG-JUL | <0.001 |
|  | FL-U-WG-JUN\|FA-M-LG-JUL | FA-M-LG-JUL\|FA-M-LG-AUG | <0.001 |
|  | FA-M-LG-JUL\|FA-M-LG-AUG | FA-M-LG-AUG\|FA-M-LG-SEP | <0.001 |
|  | FA-M-LG-AUG\|FA-M-LG-SEP | FA-M-LG-SEP\|PH-M-LG-NOV | <0.001 |
|  | FA-M-LG-SEP\|PH-M-LG-NOV | PH-M-LG-NOV\|MH-M-LG-JAN | <0.001 |

**Supplementary Table 4 Permutational multivariate analysis of variance (PERMANOVA, permutation number = 999) for 25 pairs of gut microbiome groups in terms of beta diversity indices.**

**Supplementary Table 5 The chi-square (*Χ*^2^) tests on proportions of ASV categories between contemporary gut microbiomes. WG microbiomes were integrated in the analyses of SG and LG microbiomes, but female samples were excluded. The ASV numbers in the analyses of SG and LG microbiomes were 5274 and 6294, respectively.**

| **Group 1 from SG microbiomes** | **Group 2 from LG microbiomes** | ***P*(*Χ*^2^)** |
| --- | --- | --- |
| TH-U-WG-JUN | TH-U-WG-JUN | 0.001 |
| TF-U-WG-JUN | TF-U-WG-JUN | 0.020 |
| FL-U-WG-JUN | FL-U-WG-JUN | 0.020 |
| FA-M-SG-JUL | FA-M-LG-JUL | <0.001 |
| FA-M-SG-AUG | FA-M-LG-AUG | <0.001 |
| FA-M-SG-SEP | FA-M-LG-SEP | <0.001 |
| PH-M-SG-NOV | PH-M-LG-NOV | <0.001 |
| MH-M-SG-JAN | MH-M-LG-JAN | <0.001 |

**Supplementary Table 6 The chi-square (*Χ*^2^) tests on proportions of ASV fluctuation types between contemporary gut microbiomes. WG microbiomes were integrated in the analyses of SG and LG microbiomes, but female samples were excluded. The ASV numbers in the analyses of SG and LG microbiomes were 5274 and 6294, respectively.**

| **Group 1 from SG microbiomes** | **Group 2 from LG microbiomes** | ***P*(*Χ*^2^)** |
| --- | --- | --- |
| TH-U-WG-JUN\|TF-U-WG-JUN | TH-U-WG-JUN\|TF-U-WG-JUN | 0.131 |
| TF-U-WG-JUN\|FL-U-WG-JUN | TF-U-WG-JUN\|FL-U-WG-JUN | 0.556 |
| FL-U-WG-JUN\|FA-M-SG-JUL | FL-U-WG-JUN\|FA-M-LG-JUL | <0.001 |
| FA-M-SG-JUL\|FA-M-SG-AUG | FA-M-LG-JUL\|FA-M-LG-AUG | <0.001 |
| FA-M-SG-AUG\|FA-M-SG-SEP | FA-M-LG-AUG\|FA-M-LG-SEP | <0.001 |
| FA-M-SG-SEP\|PH-M-SG-NOV | FA-M-LG-SEP\|PH-M-LG-NOV | <0.001 |
| PH-M-SG-NOV\|MH-M-SG-JAN | PH-M-LG-NOV\|MH-M-LG-JAN | <0.001 |

**Supplementary Table 7 The topological attributes for co-occurrence networks of eight gut microbiome groups. The Zi-Pi values of vertex were used to identify the types, i.e., Connectors (key nodes between modules), Module hubs (key nodes in modules), Network hubs (key nodes of whole network), and Others.**

| **Attributes** | **WG** | **M_JUL** | **M_AUG** | **M_SEP** | **M_NOV** | **M_JAN** | **M_SG** | **M_LG** |
| --- | --- | --- | --- | --- | --- | --- | --- | --- |
| **Vertex (Connectors)** | 47 | 19 | 0 | 5 | 0 | 0 | 0 | 17 |
| **Vertex (Module hubs)** | 0 | 0 | 1 | 1 | 0 | 0 | 1 | 1 |
| **Vertex (Network hubs)** | 0 | 0 | 0 | 0 | 0 | 0 | 0 | 0 |
| **Vertex (Others)** | 43 | 140 | 163 | 183 | 123 | 70 | 66 | 222 |
| **Edge** | 1763 | 483 | 294 | 724 | 266 | 244 | 114 | 648 |
| **Average degree** | 39.18 | 6.08 | 3.59 | 7.66 | 4.33 | 6.97 | 3.40 | 5.40 |
| **Average path length** | 1.24 | 2.94 | 4.69 | 3.43 | 4.15 | 1.01 | 1.72 | 3.07 |
| **Network diameter** | 4 | 8 | 10 | 13 | 12 | 2 | 4 | 9 |
| **Clustering coefficient** | 0.78 | 0.40 | 0.57 | 0.51 | 0.56 | 0.99 | 0.37 | 0.38 |
| **Density** | 0.44 | 0.04 | 0.02 | 0.04 | 0.04 | 0.10 | 0.05 | 0.02 |
| **Heterogeneity** | 0.58 | 0.97 | 0.64 | 1.10 | 0.66 | 0.90 | 0.84 | 0.94 |
| **Centralization** | 0.36 | 0.15 | 0.04 | 0.15 | 0.07 | 0.15 | 0.16 | 0.10 |
| **Modularity** | 0.11 | 0.49 | 0.84 | 0.45 | 0.75 | 0.58 | 0.63 | 0.59 |

† WG: whole gut microbiomes in June; M_JUL: male gut microbiomes in July; M_AUG: male gut microbiomes in August; M_SEP: male gut microbiomes in September; M_NOV: male gut microbiomes in November; M_JAN: male gut microbiomes in January; M_SG: male small gut microbiomes; M_LG: male large gut microbiomes.

**Supplementary Table 8 The key nodes in co-occurrence networks of eight gut microbiome groups.** † WG: whole gut microbiomes in June; M_JUL: male gut microbiomes in July; M_AUG: male gut microbiomes in August; M_SEP: male gut microbiomes in September; M_NOV: male gut microbiomes in November; M_JAN: male gut microbiomes in January; M_SG: male small gut microbiomes; M_LG: male large gut microbiomes.
